# Independently paced Ca^2+^ oscillations in progenitor and differentiated cells in an *ex vivo* epithelial organ

**DOI:** 10.1242/jcs.260249

**Published:** 2022-07-19

**Authors:** Anna A. Kim, Amanda Nguyen, Marco Marchetti, XinXin Du, Denise J. Montell, Beth L. Pruitt, Lucy Erin O'Brien

**Affiliations:** 1 Department of Molecular and Cellular Physiology, Stanford University School of Medicine, Stanford, CA 94305, USA; 2 Departments of Mechanical Engineering and Biomolecular Science and Engineering, University of California, Santa Barbara, CA 93106, USA; 3 Department of Materials Science and Engineering, Uppsala University, 75103 Uppsala, Sweden; 4 Department of Molecular, Cellular, and Developmental Biology, University of California, Santa Barbara, CA 93106, USA; 5 Department of Oncological Sciences, Huntsman Cancer Institute, University of Utah, Salt Lake City, UT 84112, USA; 6 Center for Computational Biology, Flatiron Institute, New York, NY 10010, USA; 7 Chan-Zuckerberg Biohub, San Francisco, CA 94158, USA

**Keywords:** *Drosophila*, Calcium, Epithelial cell, Live imaging, Midgut, Stem cell

## Abstract

Cytosolic Ca^2+^ is a highly dynamic, tightly regulated and broadly conserved cellular signal. Ca^2+^ dynamics have been studied widely in cellular monocultures, yet organs *in vivo* comprise heterogeneous populations of stem and differentiated cells. Here, we examine Ca^2+^ dynamics in the adult *Drosophila* intestine, a self-renewing epithelial organ in which stem cells continuously produce daughters that differentiate into either enteroendocrine cells or enterocytes. Live imaging of whole organs *ex vivo* reveals that stem-cell daughters adopt strikingly distinct patterns of Ca^2+^ oscillations after differentiation: enteroendocrine cells exhibit single-cell Ca^2+^ oscillations, whereas enterocytes exhibit rhythmic, long-range Ca^2+^ waves. These multicellular waves do not propagate through immature progenitors (stem cells and enteroblasts), of which the oscillation frequency is approximately half that of enteroendocrine cells. Organ-scale inhibition of gap junctions eliminates Ca^2+^ oscillations in all cell types – even, intriguingly, in progenitor and enteroendocrine cells that are surrounded only by enterocytes. Our findings establish that cells adopt fate-specific modes of Ca^2+^ dynamics as they terminally differentiate and reveal that the oscillatory dynamics of different cell types in a single, coherent epithelium are paced independently.

## Abstract (precis)

**Summary:** Live imaging of the *Drosophila* intestinal epithelium reveals that stem cells and their terminal progeny exhibit distinct, fate-specific Ca^2+^ oscillations that are paced independently of each other.

## INTRODUCTION

Ca^2+^ is a versatile signaling molecule that regulates vital cellular functions such as contraction and cellular excitability in all organ systems (Berridge et al., 2000; Carafoli, 2002; Clapham, 2007). Changes in Ca^2+^ signal dynamics have been linked to crucial cell behaviors, such as intercellular communication, cell cycle, proliferation and migration (Hofer et al., 2000; Humeau et al., 2018; Wei et al., 2009; Xu et al., 2017). Intracellular Ca^2+^ concentrations can also regulate cellular responses and physiology by modulating signal transduction pathways such as MAPK (Apáti et al., 2003; Kupzig et al., 2005) and inositol trisphosphate (IP_3_) (Harootunian et al., 1991; Sjaastad et al., 1996; Taylor and Thorn, 2001). In excitable tissues, such as in electrically coupled cells of heart muscle, Ca^2+^ plays a central role in propagating the impulse that coordinates the pacing of contractions (Cheng et al., 1996; Dewenter et al., 2017). Large-scale Ca^2+^ waves have been observed in cultured astrocytes and in mouse hippocampus astrocyte networks (Innocenti et al., 2000; Kuga et al., 2011).

Although Ca^2+^ dynamics have been studied widely in cell culture, investigations in tissues and organs – particularly those that are non-excitable – have been more limited. Studies in *Drosophila* demonstrated intercellular Ca^2+^ waves that traverse large tissue domains and might depend on actomyosin organization (Balaji et al., 2017). These tissue-level Ca^2+^ dynamics, which occur in imaginal discs, the tightly coupled epithelial structures that give rise to the external structures of the adult fly, were implicated in organ growth and size modulation (Brodskiy et al., 2019; Soundarrajan et al., 2021 preprint). Furthermore, intercellular Ca^2+^ waves were shown to be induced mechanically in these developing epithelia (Narciso et al., 2017). Finally, blood progenitors in the *Drosophila* lymph gland were recently shown to form a gap-junction-mediated network that can regulate Ca^2+^ signaling (Ho et al., 2021).

By contrast, little work has been done to examine Ca^2+^ dynamics in non-excitable tissues composed of heterogenous cell types. The question of whether and, if so, how tissue-scale Ca^2+^ oscillations are coordinated between different cell types is particularly intriguing for stem-cell-based tissues that undergo constitutive cellular turnover. As the fates of individual stem-cell daughters change during differentiation, any fate-associated differences in Ca^2+^ oscillations must evolve as fate decisions are made.

Here, we establish the adult *Drosophila* intestine as an *ex vivo* model for studying diverse Ca^2+^ dynamics that occur simultaneously in different cell types in a self-renewing mature organ. The midgut, like most mature organs, undergoes continuous turnover in which tissue-specific stem-cell divisions produce progeny that differentiate into multiple cell types. Ca^2+^ signaling has been established as a key regulator of midgut stem cell activity (Deng et al., 2015; He et al., 2018). Ca^2+^ transients are mechanically induced via the mechanosensitive ion channel Piezo in a subpopulation of stem cells in the fly gut (He et al., 2018). However, how Ca^2+^ dynamics regulate and are regulated by cell differentiation and organ-scale inputs remains largely unknown.

Here, we simultaneously expressed spectrally distinguishable Ca^2+^ indicators in each midgut cell type and performed real-time analysis of single- and multi-cell oscillations to produce a fate-resolved, tissue-scale overview of Ca^2+^ dynamics. We investigated fate-specific changes as cells differentiated in their native tissue environment and describe rhythmic multicellular Ca^2+^ waves in enterocytes and cell-type-specific Ca^2+^ oscillations. These results demonstrate that as cells differentiate from stem-cell-like into distinct terminal fates, they adopt cell-type-specific Ca^2+^ oscillations and waves that are a hallmark of the mature organ.

## RESULTS

### All major cell types in the adult middle midgut exhibit Ca^2+^ oscillations under whole-organ culture *ex vivo*

**Fig. 1.**
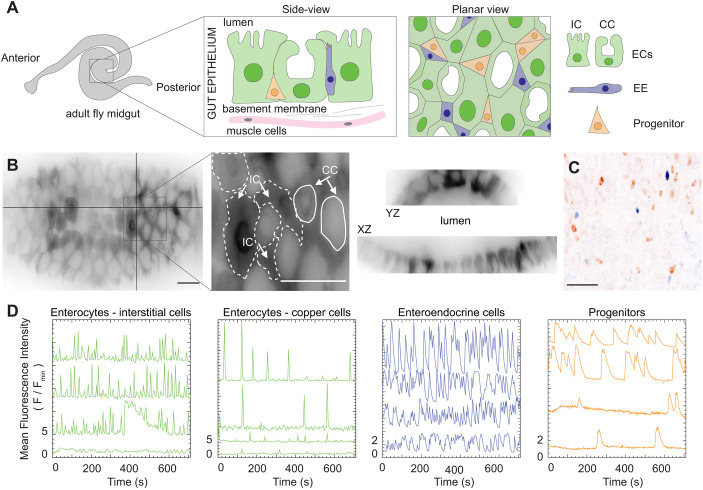
**Distinct Ca^2+^ oscillations in the major cell types – enterocytes, enteroendocrine cells, and progenitors – of the middle region of the midgut.** (A) Schematic of the middle (R3) midgut, the copper cell region, with side and planar views. Interstitial cell, IC; copper cell, CC; enterocyte, EC; enteroendocrine cell, EE. (B) Maximum-intensity projection (left) and orthogonal views (right) of a *z*-stack acquired with a light-sheet microscope (*mex-GAL4>UAS-GCaMP6s*). Interstitial and copper cells were distinguished by the unique shape of the copper cells (middle); examples are marked with white line overlays and arrows in the inset. Estimates for cell boundaries between ICs are indicated by the dashed lines. (C) Maximum-intensity projection demonstrating the distribution of enteroendocrine cells (blue) and progenitors (orange) (*esg-LexA>LexAop-jRCaMP1b*, *pros-GAL4>UAS-GCaMP6s*) using an inverted colormap (Leterrier; see https://github.com/cleterrier/ChrisLUTs). (D) Stem-cell daughters acquire distinct patterns of Ca^2+^ oscillations. Representative traces of fluorescence intensity of genetically encoded Ca^2+^ indicators in enterocytes (*mex-GAL4>UAS-GCaMP6s*, Movie 1), enteroendocrine cells (*prosGAL4>UAS-GCaMP6s*, Movie 2) and progenitors (*esg-GAL4>UAS-jRCaMP1b*, Movie 3). Representative traces were selected from five movies for ECs, five movies for EEs and four movies for progenitors. Scale bars: 25 µm.

To examine Ca^2+^ dynamics in the adult fruit fly midgut, we performed live imaging of whole organs employing the genetically encoded Ca^2+^ sensors, GCaMP6s (Chen et al., 2013) and jRCaMP1b (Dana et al., 2016), hereafter referred to as GCaMP and RCaMP, respectively. To examine Ca^2+^ oscillations in each midgut cell type (Fig. 1A–C), we expressed a genetically encoded Ca^2+^ sensor using the GAL4/UAS system (Brand and Perrimon, 1993) under the control of cell-type-specific drivers. We dissected midguts from mated female fruit flies aged 4–7 days and mounted them by adapting an *ex vivo* organ culture protocol (Marchetti et al., 2021 preprint) (see Materials and Methods). To characterize cell-type-specific Ca^2+^ activity in terms of temporal dynamics, we traced the mean fluorescence intensity of individual cells as a function of time (see Materials and Methods) and quantified the frequency of oscillations.

### Terminally differentiated cells of the R3 midgut exhibit distinct Ca^2+^ dynamics

The R3 region of the midgut, which is responsible for acid secretion, attracted our attention due to the consistent appearance of rapid, multicellular Ca^2+^ waves that travel through R3 enterocytes. We also observed Ca^2+^ dynamics in enterocytes in the posterior region of the midgut, but not consistently. We did not observe any Ca^2+^ dynamics in the anterior (R2) region, which might reflect a physiological difference in this region of the gut, or which might be a consequence of dissection or *ex vivo* culture. We used the GAL4 driver *midgut expression 1* or *mex1* (*mex-GAL4*) to label enterocytes and focused the remainder of our experiments on R3.

As a starting point, we measured the frequency of Ca^2+^ oscillations in single cells from each cell type over time. Enterocytes in the R3 region are subdivided into acid-secreting copper cells (CCs) and interstitial cells (ICs) (Dubreuil, 2004; Poulson and Bowen, 1952; Strasburger, 1932), which we distinguished by the unique shape of copper cells (Fig. 1B).

Interstitial cells exhibited wave-like Ca^2+^ dynamics with a mean oscillation frequency of 31±8.9 mHz (s.e.m.) per gut. By comparison, copper cells primarily displayed Ca^2+^ spikes at a frequency of 5.2±0.9 mHz per gut (Fig. 1D).

Interestingly, we also observed relatively rapid fluorescence oscillations in enteroendocrine cells [*prospero* (*pros*)-expressing] of 15±4.3 mHz per gut, approximately twice that of progenitors [stem cells and enteroblasts, which both express *escargot* (*esg*)], 7.1±2.6 mHz per gut. Finally, the robust Ca^2+^ oscillations we observed in progenitors are consistent with prior reports (Deng et al., 2015; He et al., 2018). The complete dataset of the average oscillation frequencies per midgut, including and excluding non-oscillating cells for all cell types, is included in Tables S2 and S3.

### Long-range Ca^2+^ waves travel across enterocytes

**Fig. 2.**
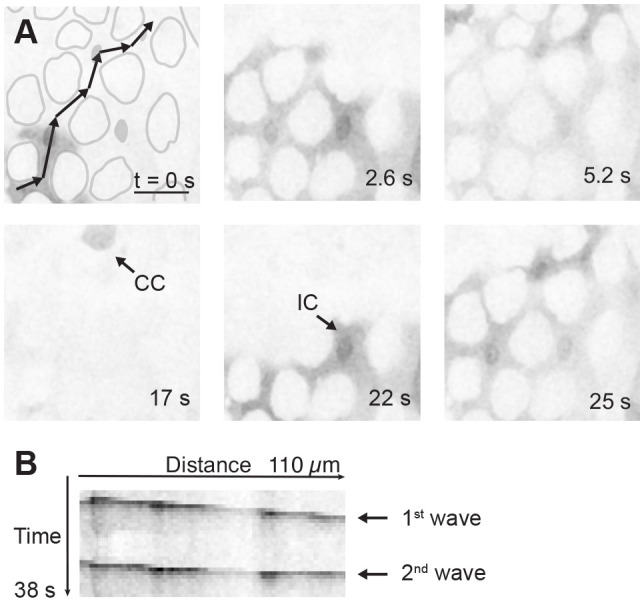
**Ca^2+^ wave propagation in enterocytes.** (A) Propagation of Ca^2+^ waves across several cell lengths (*mex-GAL4>UAS-GCaMP6s*) in a single plane. Approximations of copper cell outlines are depicted in gray. Single black arrows (bottom left and middle) identify examples of copper cells (CCs) and interstitial cells (ICs) based on the unique shape of copper cells. The trajectory of a Ca^2+^ wave (identified visually) is depicted by the linked black arrows (top left). Scale bar: 25 µm. See Movie 1. (B) Kymograph along the direction of the Ca^2+^ wave identified in A, demonstrating that a second wave appears and propagates in a similar direction (arrows). Images are representative of Ca^2+^ waves in five experiments.

Unexpectedly, we found long-range Ca^2+^ waves that propagate across large fields of enterocytes in the R3 region of the midgut (Fig. 2A). We observed high GCaMP signals that shifted rapidly across several cell lengths in multiple directions (Movie 1), with the longest wave we observed covering approximately five cells. These Ca^2+^ waves, which exhibited a multitude of dynamic patterns, such as propagating along a single trajectory, splitting and colliding, did not have an obvious point of origin. Multi-enterocyte waves traveled almost exclusively through interstitial cells and rarely through copper cells. GCaMP signals appeared repeatedly in the same cell over time as part of multi-directional waves that travelled through the tissue (Fig. 2B). In this example (Movie 1), individual interstitial cells spiked, on average, every 23 s, leading to an average frequency of 44±1.4 mHz.

**Fig. 3.**
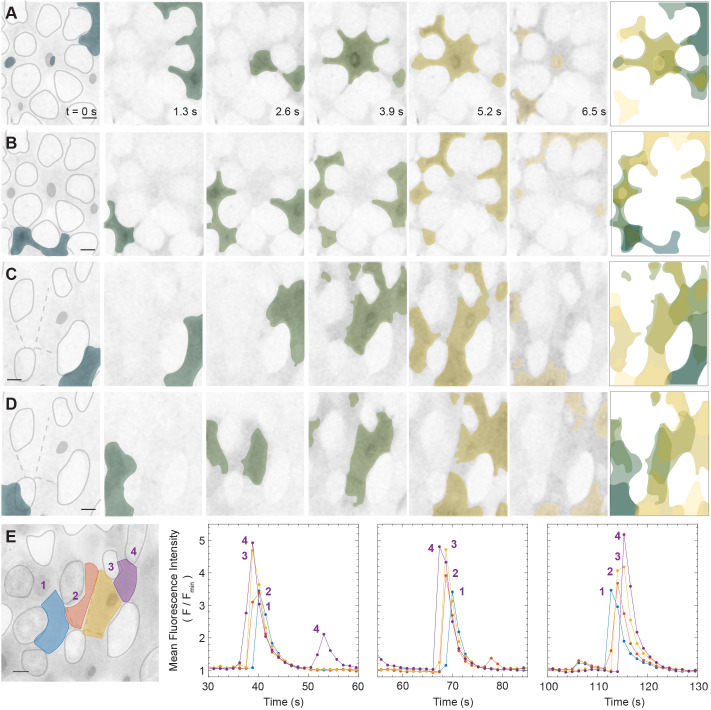
**Ca^2+^ waves in interstitial cells, identified as the space between copper cells, exhibit a multitude of dynamic patterns.** (A–D) Ca^2+^ waves in two separate fields from the same midgut are shown, demonstrating diverse spatial propagation dynamics in the same tissue. Approximations of copper cell outlines are depicted in gray. False coloring of the Ca^2+^ signal is done using the scientific colormap bamako (https://www.fabiocrameri.ch/colourmaps/; Crameri et al., 2020). The signals transition from a dark to light color as a function of time, with the last image illustrating a temporal maximum projection of the false colors. (E) Four adjacent regions of interest (ROIs) with corresponding normalized mean fluorescence intensity of GCaMP as a function of time (*mex-GAL4>UAS-GCaMP*). Maximum intensity projections are shown. Images are representative of ten Ca^2+^ waves observed in the same gut. Scale bars: 10 µm. See Movie 1.

To assess whether individual waves traveled along recurring paths, we followed GCaMP signals in selected regions of a single midgut (Fig. 3A–D), which encompassed eight to 13 cells in an area of ∼3000 µm^2^. Based on the fluorescence intensity, we estimated cell outlines and identified copper cells based on their unique cell shapes and signals (Fig. 1B); interstitial cells were identified as the space between the copper cells (Dubreuil, 2004; Shanbhag and Tripathi, 2009). The waves did not, to the extent of our observation, exhibit a predictable pattern, even when they recurred in the same region. This is exemplified in Fig. 3E, where we traced the mean fluorescence intensity as a function of time in four adjacent regions of interest (ROIs), across approximately 50 µm in width from anterior to posterior. Over a 100 s interval, there were three large increases in the Ca^2+^ signal (∼3- to 5-fold above the baseline) and the signal traveled in both proximal-distal and distal-proximal directions.

### Ca^2+^ oscillations in progenitor cells neither propagate from Ca^2+^ waves in enterocytes nor correlate with oscillations in enteroendocrine cells

**Fig. 4.**
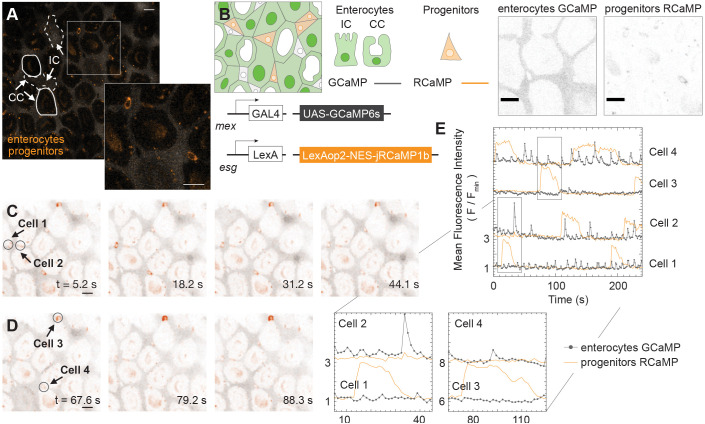
**Ca^2+^ dynamics are independent between enterocytes and progenitors.** An analysis of results from Movie 4. (A) An image from a single-plane 240 s movie. Examples of enterocyte cell outlines are approximated and depicted in white. Cells are distinguishable for enterocytes (gray) and progenitors (orange pseudocolor). The lower right image shows a 2× magnification of the region within the square. (B) Genetic design of the dual reporter line (*mex-GAL4>UAS-GCaMP*, *esg-LexA>LexAop-jRCaMP*). Cell types are distinguished by their expression of fluorescent markers: enterocytes (GCaMP) and progenitors (RCaMP). (C,D) Time-lapse sequences from the single-plane movie showing Ca^2+^ dynamics simultaneously in enterocytes and progenitors. ROIs were selected to encompass single progenitors (circles) and to compensate for its movement throughout the movie. Images are representative of ten ROIs total in this movie. (E) Mean fluorescence intensity of GCaMP (gray) and RCaMP (orange) as a function of time for the entire movie with insets (bottom) that contain the time-lapse sequences shown in C and D for the identified four progenitor cells. Scale bars: 10 µm.

To directly test whether Ca^2+^ oscillations in distinct cell types are coupled, we used the orthogonal driver systems *LexA/LexAop* (Lai and Lee, 2006; Szüts and Bienz, 2000) and *GAL4/UAS* (Brand and Perrimon, 1993) to express spectrally differentiable Ca^2+^ sensors in two cell types simultaneously, then we performed multi-channel imaging to record the signal from both. We labeled progenitor cells using the insertion *StanEx^SJH1^*, from the StanEx collection of *LexA*-based enhancer trap drivers (Kockel et al., 2016), in which the LexA transcription factor is inserted upstream of the transcription start site for *escargot.* For convenience, we refer to this insertion as *esg-LexA* (Fig. 4A,B; Figs S1 and S2). In combination with *mex-GAL4*, we could visualize Ca^2+^ dynamics directly in enterocytes and progenitors, simultaneously (Fig. 4C,D; Movie 4).

We selected ROIs to cover a region a little larger than a single progenitor (Fig. 4A), so that the cell of interest remained within the ROI even as the gut shifted due to the contracting muscle cells. Mean fluorescence intensities of the ROIs were traced as a function of time for both GCaMP (enterocyte) and RCaMP (progenitor) channels (Fig. 4E). We illustrate the feasibility of using the orthogonal driver systems to simultaneously image Ca^2+^ dynamics in interstitial cells and progenitors. Characteristic oscillations of ∼24 mHz were observed for interstitial cells in the GCaMP channel and wide signal elevations (20 s) in the RCaMP channel; these were comparable to observations when using a single-driver system (Fig. 1D).

To quantitatively assess whether Ca^2+^ oscillations of progenitors and enterocytes were correlated, we analyzed the four ROIs (Fig. 4E) that covered progenitor cells 1–4 and their juxtaposed enterocytes (direct enterocyte-progenitor pairs), by applying a formula for cross correlations between two time series with time lag h (see Table S4, cross correlation with time lag for finite time series). With time lag 0, which corresponds to the Pearson correlation, the enterocyte-to-progenitor cross correlations for the four direct enterocyte-progenitor pairs in the ROIs (enterocyte 1 to progenitor 1, enterocyte 2 to progenitor 2, etc.) have absolute values <0.2 for all four direct pairs (these four correlation values are approximately 0.064, 0.032, −0.10 and 0.19), indicating weak correlation. When all non-zero time lags are considered, the cross correlations for direct enterocyte-progenitor pairs never exceed an absolute value of 0.32 (Fig. S4). These values do not support the hypothesis that progenitor and enterocyte oscillations are correlated.

We also considered cross correlations between enterocyte-progenitor pairs in which the enterocyte and the progenitor are in different ROIs. Table S4 reports all the correlation values for all direct and non-direct pairs for time lags up to 12 s.

We also used the combination of the *GAL4/UAS* and *LexA/LexAop* systems to simultaneously visualize Ca^2+^ dynamics in enteroendocrine cells (*pros-GAL4>UAS-GCaMP*) and progenitors (*esg-LexA>LexAOp-jRCaMP*). Similar to enterocytes and progenitors, we observed that Ca^2+^ oscillations in enteroendocrine cells and progenitor cells were independent of each other (Movie 5).

### Propagation of Ca^2+^ waves and oscillatory Ca^2+^ dynamics depend on functional gap junctions

**Fig. 5.**
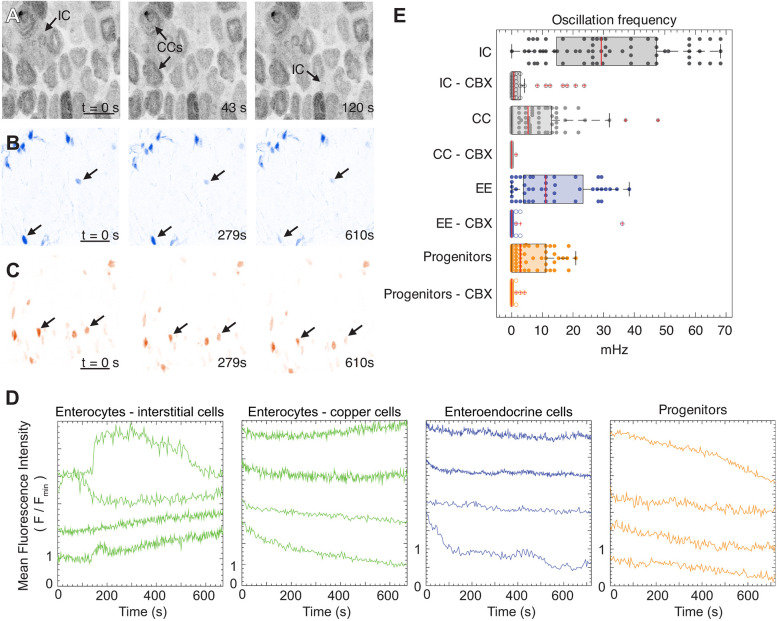
**Propagation of Ca^2+^ waves and oscillatory Ca^2+^ dynamics depend on functional gap junctions.** Time-lapse image sequences of maximum-intensity projections for tissues incubated with the gap junction inhibitor carbenoxolone (CBX). (A) Enterocytes (*mex-GAL4>UAS-GCaMP*, Movie 6) – interstitial cells (ICs) and copper cells (CCs), (B) enteroendocrine cells (EE) (*pros-GAL4>UAS-GCaMP*, Movie 7) and (C) progenitors (*esg-GAL4>UAS-jRCaMP1b*, Movie 8) are indicated by black arrows. Scale bars: 25 µm. (D) Representative traces of fluorescence intensity of genetically encoded Ca^2+^ indicators were selected from four movies for ECs, five movies for EEs and three movies for progenitors. (E) Comparison of Ca^2+^ oscillation frequency for the different cell types in the middle midgut region with and without the gap junction inhibitor carbenoxolone (CBX). Boxes represent the 25–75th percentiles, whiskers extend to the 75th percentile plus 1.5× interquartile range and 25th percentile minus 1.5× interquartile range, and the median is marked with a red line. The total number of ROIs analyzed per condition were as follows: IC, 65 (5 guts); IC – CBX, 38 (4 guts); CC, 74 (5 guts); CC – CBX, 44 (4 guts); EE, 45 (5 guts); EE – CBX: 51 (5 guts); progenitors, 59 (4 guts); progenitors – CBX: 39 (3 guts).

Gap junctions are intercellular channels that allow direct transfer of small molecules and ions. Thus, cells sharing gap junctions are electrically coupled. For example, in the regenerative basal layer of the skin epithelium, directed Ca^2+^ signaling is regulated by a major gap junction protein (Moore et al., 2021 preprint). As the Ca^2+^ waves we observed appeared to traverse several cell lengths, we hypothesized that Ca^2+^ ions propagate across cells via gap junctions. To examine this hypothesis, we blocked gap junctions by adding the small-molecule inhibitor carbenoxolone (CBX) (Balaji et al., 2017; Spéder and Brand, 2014) to the imaging medium for 15 min prior to and during imaging midguts with cell-type-specific expression of Ca^2+^ indicators. We analyzed the fluorescence intensity of Ca^2+^ indicators in individual cells as a function of time for each cell type in control and CBX-treated guts (Fig. 5D,E; Movies 6–8).

We found that gap junction inhibition sharply inhibited Ca^2+^ oscillations for all cell types, but the consequence to cytosolic Ca^2+^ levels differed depending on the cell type. Although interstitial cells exhibited weaker GCaMP signals with CBX treatment, copper cells exhibited substantially higher GCaMP signals (Fig. 5A). One possible explanation for this unexpected result is that copper cells might accumulate cytosolic Ca^2+^, perhaps from extracellular sources, when Ca^2+^ flux through interstitial cells is blocked.

CBX treatment eliminated multicellular Ca^2+^ waves that normally propagate through interstitial cells. CBX treatment also abrogated Ca^2+^ oscillations in enteroendocrine cells (Fig. 5B) and progenitors (Fig. 5C). As the oscillations of progenitors were not coupled to enterocytes (Fig. 4), this result was unexpected; potentially, whole-organ inhibition of gap junctions by CBX could disrupt organ-scale Ca^2+^ homeostasis with consequent inhibition of single-cell oscillations. Consistent with this notion, Ca^2+^ oscillations in the intestinal stem cells have been shown to depend on both the influx of Ca^2+^ ions through the plasma membrane and on the internal release of Ca^2+^ ions that have been actively sequestered in the intracellular stores by sarco-endoplasmic reticulum Ca^2+^-ATPase (SERCA) (Deng et al., 2015).

## DISCUSSION

Examining Ca^2+^ dynamics in the midgut of adult *Drosophila*, we found that differently fated cells in the midgut R3 region exhibited characteristic patterns of Ca^2+^ oscillations. Performing live imaging of midguts *ex vivo*, we identified propagation of Ca^2+^ waves through networks of interstitial cells and characterized Ca^2+^ oscillations in enteroendocrine cells and progenitors. Employing orthogonal expression of red and green Ca^2+^ sensors, we demonstrated that the Ca^2+^ dynamics of enterocytes and progenitors are paced independently of one another. We also found that the Ca^2+^ dynamics of progenitors and enteroendocrine cells are paced independently. Our work demonstrated that whole-organ inhibition of gap junctions eliminated dynamic Ca^2+^ responses in all three cell types and led to an accumulation of Ca^2+^ ions in copper cells. Thus, gap junction inhibition potentially disrupts organ-scale Ca^2+^ homeostasis.

An intriguing aspect of our results is that differentiated cell types in the same epithelium, despite being derived from the same progenitor cell population, exhibit Ca^2+^ dynamics that differ from their mother cells and from each other. This finding implies that as stem-cell progeny undergo differentiation, they adopt fate-specific modes of Ca^2+^ pacing. Ca^2+^ signal integration remains an intriguing open question due to its pleiotropic and ubiquitous nature. One possibility is that unique oscillatory Ca^2+^ patterns are interpreted by downstream effectors to subsequently activate different cellular processes; another is that the Ca^2+^ dynamics support the physiological functions of the specific cell types.

What are these cell-type-specific physiological functions? Ca^2+^ oscillations in stem cells can influence division rate (Deng et al., 2015), whereas in terminal progenitors they regulate enteroendocrine differentiation (He et al., 2018). The functions of Ca^2+^ signaling in mature enteroendocrine cells and enterocytes remain to be elucidated, but several studies in developing tissues and in cultured cells raise attractive possibilities. For example, in developing epithelial tissues, Ca^2+^ waves and spikes have been implicated in organizing actomyosin networks (Balaji et al., 2017; Hoyer et al., 2020 preprint; Ready and Chang, 2021), remodeling and repair of damaged occluding junctions (Varadarajan et al., 2022), responding to exogenous mechanical loads (Narciso et al., 2017) and coordinating growth at the tissue scale (Brodskiy et al., 2019). It is conceivable that Ca^2+^ waves in midgut enterocytes have similar roles, as enterocytes must maintain cytoskeletal networks and occluding-junction-based barrier function during continual mechanical compression due to peristalsis. Another fascinating possibility comes from studies of the *Caenorhabditis elegans* intestine, which identified gap-junction-mediated Ca^2+^ waves in intestinal epithelial cells as essential for coordinating rhythmic defecation (Espelt et al., 2005; Peters et al., 2007; Teramoto and Iwasaki, 2006). Finally, in cultured cells, the frequency of Ca^2+^ oscillations and spikes has been shown to control differential activation of transcription factors (Dolmetsch et al., 1997; 1998; Li et al., 1998). It is conceivable that the distinct frequencies of Ca^2+^ oscillations in midgut enterocytes, enteroendocrine cells and/or progenitors are tuned to regulate the activity of cell-type-specific transcription factors.

In the midgut, as in other multicellular systems, gap junctions are crucial for Ca^2+^ oscillations (Leybaert and Sanderson, 2012). These cell-cell junctions are oligomers of connexins (vertebrates) or innexins (invertebrates), with eight innexin genes identified in *Drosophila* that encode at least ten transmembrane proteins (Adams et al., 2000; Stebbings et al., 2002). Bulk transcriptomic analysis of the R3 region indicates that enterocytes, enteroendocrine cells and progenitors express *innexin7* (*Inx7*) and, to a lesser extent, *innexin2* (*Inx2*) (Dutta et al., 2015); single-cell transcriptomic data also reveal the expression of *innexin7* and *innexin2* in enterocytes, but suggest that *innexin3* (*Inx3*) is the predominant innexin in progenitor and enteroendocrine cells (Hung et al., 2020; Li et al., 2022). Future work to establish the molecular composition and connectivity of gap junctions in the midgut epithelium will be invaluable for understanding the regulation and functions of Ca^2+^ dynamics.

In summary, our findings establish a novel model for studying cell-type-specific Ca^2+^ dynamics within a heterogeneous population of stem and differentiated cells in an adult tissue. Our observations lay the groundwork for using this highly tractable genetic model to investigate the roles of spatiotemporal Ca^2+^ changes within signal transduction, organ renewal and stem cell differentiation.

## MATERIALS AND METHODS

### Fly stocks

We obtained *20XUAS-IVS-NES-jRCaMP1b-p10* (BL63793), *20XUAS-IVS-GCaMP6s* (BL42746), *13XLexAop2-IVS-NES-jRCaMP1b-p10* (BL64428) and *P{ST.lexA::HG}SJH-1* (Kockel et al., 2016) (BL66632, referred to as *esg-LexA* in this paper, Figs S1 and S2) from the Bloomington Stock Center, Indiana University, IN, USA. *esg-GAL4* was obtained from the Department of *Drosophila* Genomics and Genetic Resources (DGGR), Kyoto Institute of Technology, Japan. The following stocks were gifts: *mex-GAL4* (Carl Thummel, Howard Hughes Medical Institute University of Utah Salt Lake City, UT, USA), *esg-GFP[KI]/CyO* (Norbert Perrimon, Department of Genetics, Harvard Medical School, Boston, MA, USA) and *pros-GAL4* (Sarah Siegrist, Department of Biology, University of Virginia, Charlottesville, VA, USA) (Matsuzaki et al., 1992). The complete list of key resources can be found in Table S1.

### *Drosophila* husbandry

Flies were fed a diet of standard cornmeal molasses food at 25°C or room temperature. Flies were collected 0–24 h post eclosion, placed in vials with males and shifted to 25°C with 12 h light on and 12 h light off. The flies were fed a diet of standard cornmeal molasses food supplemented with yeast paste (Red Star, Active Dry Yeast) and the food vials were changed every 1–3 days. Experiments were performed on female flies, 4–7 days post eclosion.

### Validation of *esg-LexA*

To understand the expression of *esg-LexA*, *esg-LexA>LexAop-jRCaMP1b* was co-expressed with *esg-GAL4>UAS-his2b::CFP* and *esg-GFP* in two separate experiments. Comparison between *esg-LexA>LexAop-jRCaMP1b* and *esg-GAL4>UAS-his2b::CFP* has shown that *esg-LexA>LexAop-jRCaMP1b* almost always colocalizes with *esg-GAL4>UAS-his2b::CFP* (Matsuzaki et al., 1992). There are few cells that express *esg-GAL4>UAS-his2b::CFP*, but not *esg-LexA>LexAop-jRCaMP1b*, possibly because *LexAop-jRCaMP1b* is a transient signal. On occasion, we observed cells that express *esg-LexA>LexAop-jRCaMP1b* but not *esg-GAL4>UAS-his2b::CFP* (Fig. S1B). In general, *esg-LexA>LexAop-jRCaMP1b* exhibits a weaker signal. *esg-LexA>LexAop-jRCaMP1b* also colocalized with *esg-GFP[KI]* (*esg-GFP*) and similarly, the signal is stronger in *esg-GFP* (Matsuzaki et al., 1992). A large majority of *esg-LexA>LexAop-jRCaMP1b* cells also expressed *esg-GFP*. There are several cells that express *esg-GFP* but not *esg-LexA>LexAop-jRCaMP1b*.

### *Ex vivo* imaging

Female flies were briefly anaesthetized on carbon dioxide and then placed on ice in Eppendorf tubes to induce a chill coma. Guts were dissected in room temperature in adult hemolymph-like (AHL) medium (108 mM NaCl, 5 mM KCl, 2 mM CaCl_2_, 8.2 mM MgCl_2_, 4 mM NaHCO_3_, 1 mM NaH_2_PO_4_, 5 mM trehalose, 10 mM sucrose, 5 mM HEPES, pH 7.5, prepared by Electron Microscopy Sciences) and then bathed in whole-organ *ex vivo* culture medium with 10 µg/ml isradipine (Selleck Chemicals) to decrease muscle contractions. The guts were then transferred in a droplet to #1.5 coverslips coated with poly-L-lysine (P4832-50ML, Sigma-Aldrich) with 120 µm spacers (620,001, Grace Bio-Labs) and sealed with 250 µm-thick polydimethylsiloxane sheets (see Fig. S3). The assembled view of the *ex vivo* midgut mount for inverted microscopy is illustrated in Fig. S3.

For experiments with the gap junction inhibitor, carbenoxolone (C4790-1G, Sigma-Aldrich) was added to the *ex vivo* culture medium (100 µM CBX) and the tissues were incubated in this medium for 15 min prior to imaging.

### Composition of whole-organ *ex vivo* culture medium

The medium for *ex vivo* midgut culture was used as previously described (Marchetti et al., 2021 preprint). We supplemented a base of Schneider's Medium (21,720,024, Thermo Fisher Scientific) with 55 mM L-glutamic acid monosodium salt (AAJ6342409 Alfa Aesar, Thermo Fisher Scientific, diluted from 1 M stock prepared in Schneider's medium), 50 mM Trehalose (T5251-10G, Sigma-Aldrich, diluted from 1 M stock prepared in Schneider's medium), 2 mM N-acetyl cysteine (antioxidant that delays phototoxicity) (A9165-5G, Sigma-Aldrich, diluted from a 200 mM stock prepared in sterile water), 1 mM tri-sodium citrate (antioxidant that delays phototoxicity) (PHR1416-1G, Sigma-Aldrich, diluted from a 1 M stock prepared in Schneider's medium) and 5 mM HEPES (from 1 M solution, H0887-20 Ml, Sigma-Aldrich).

### Microscopy

An inverted Leica SP8 resonant scanning confocal microscope with a 40×/1.1 water-immersion objective was used to acquire movies that were analyzed in this study. Movies were captured at room temperature (20–23°C). Confocal stacks were acquired with a *z*-step of 2 or 4 µm and typically contained three to nine slices. For the complete list of movies and their information, including the genotypes, see Table S5. The movies and fluorescence intensity data are available on the European Molecular Biology Laboratory's European Bioinformatics Institute (EMBL-EBI) BioImage Archive under the accession number S-BSST849. Movies were captured with cycle time of 4.7 s or faster. Movies were included in data analysis (Fig. 5E) only if they were at least 290 s long. A Zeiss Z.1 Lightsheet was used with a 20×/1.0 objective by flowing a midgut into tubing (FEP009-031-B, Western Analytical) to obtain a high-resolution *z*-stack of the R3 region for Fig. 1B.

### Measurements of mean fluorescence intensity

Movie stacks were imported into Fiji (Schindelin et al., 2012) using the Bio Formats (Linkert et al., 2010) plugin. In the acquired movie stacks, we visually identified a plane that corresponded primarily to copper cells or to interstitial cells. In the selected plane, we drew ROIs manually to correspond to the cell type of interest, then we obtained mean fluorescence intensity values for each time point in Fiji. In the case of progenitors and enteroendocrine cells, we first converted *z*-stacks into maximum projection movies in Fiji. We identified cells of interest manually and then tracked the mean fluorescence intensity of cells using the Active Contours (Dufour et al., 2011) plugin in Icy (de Chaumont et al., 2012). We plotted mean fluorescence intensity values (F) relative to the minimum fluorescence values (F_min_) and peaks were identified using custom codes in MATLAB 2019b (available upon request) with the aid of the Signal Processing Toolbox. Peaks were manually verified.

### Data analysis

The average oscillations in mHz for the different cell types are presented in Tables S2 and S3, per midgut. The average oscillations are also presented if only cells exhibiting at least one oscillation are included in the analysis.

### Cross correlation with time lag for finite time series

To calculate cross correlations between enterocyte (EC) and progenitor time series data, we used the following formula for cross correlations between two time series with time lag h:

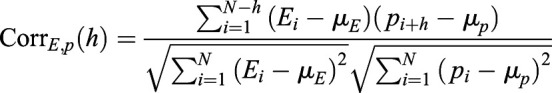
Here, the data points, E_i_ and p_i_ stand for the enterocyte and progenitor intensity at timepoint i, respectively, and h is the time lag. The quantities µ_E_ and µ_p_ refer to the mean intensity of enterocytes and progenitors over the entire time series. We use the time series data presented in Fig. 4, where we identified four ROIs of interest, with cell types distinguished by the expression of their fluorescent markers: enterocytes (GCaMP) and progenitors (RCaMP).

## Supplementary Material

Supplementary information

Reviewer comments
