# Supplementary material for: Independently paced Ca2+ oscillations in progenitor and differentiated cells in an ex vivo epithelial organ
Source: J Cell Sci. 2022 Jul 19;135(14):jcs260249. doi: 10.1242/jcs.260249 (PMC9450890; doi:10.1242/jcs.260249)
Supplement: Supplementary information [file joces-135-260249-s1.pdf]

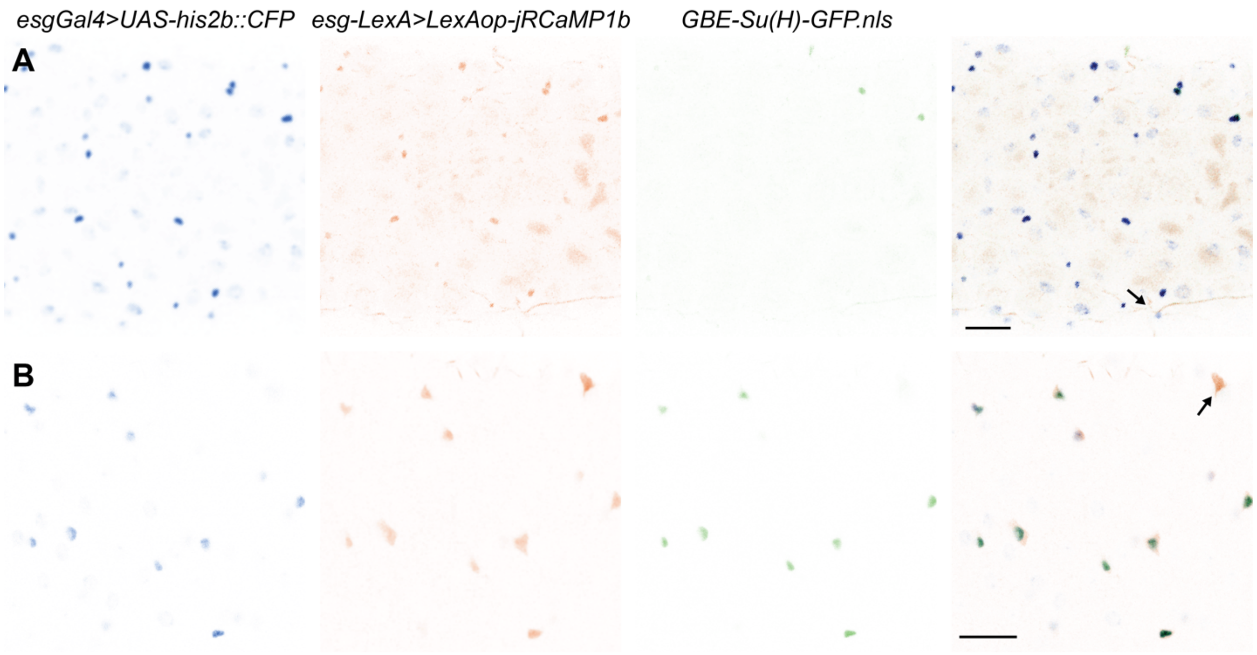

Fig. S1. Co-expression of esgGAL4>UAS-his2b::CFP and esg-LexA>LexAop-jRCaMP1b in the A) middle midgut region and the B) posterior midgut. Few cells express GBE-Su(H)-GFP in the middle midgut compared to the posterior. The arrow in A) highlights an example of a cell that expresses esgGAL4>UAS-his2b::CFP but not esg-LexA>LexAop-jRCaMP1b and in B) the opposite. All scale bars, 25 µm.

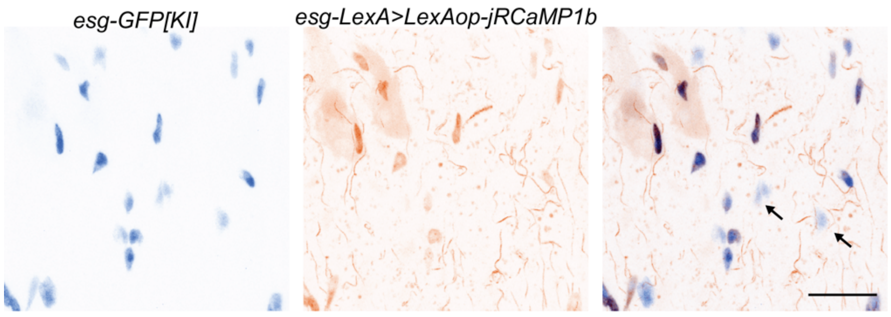

Fig. S2. Co-expression of esg-GFP and esg-LexA>LexAop-jRCaMP1b in the copper cell region. The arrows highlight examples of cells that express esg-GFP but not esg-LexA>LexAop-jRCaMP1b. All scale bars, 25 µm.

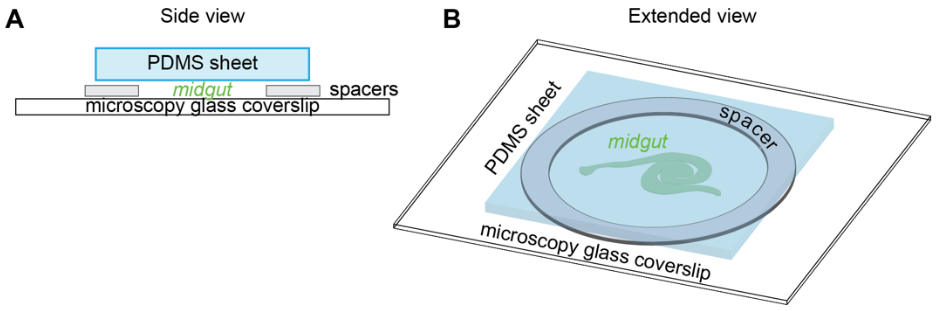

Fig. S3. Schematic of the ex vivo midgut mount for inverted microscopy. Spacer with a double-sided adhesive was attached to a microscopy coverslip coated with poly-l-lysine. The midgut was gently placed at the center in a drop of whole organ ex vivo culture medium, adapted from (Marchetti, Zhang and Edgar, 2021). PDMS sheet was gently attached to the spacer. A) Side view and B) extended view.

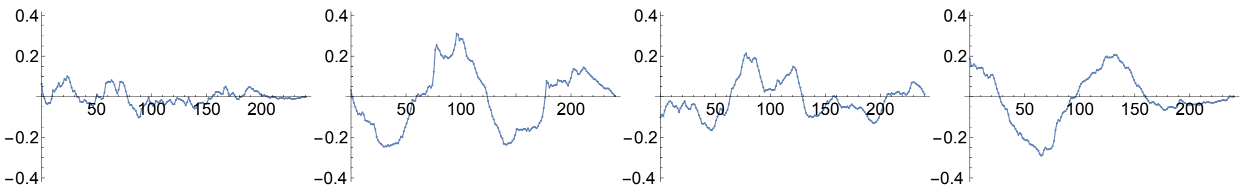

Fig. S4. Plots for the four direct EC progenitor pairs for time lags up to the full length of the time series, with time lag (s) on the x-axis and correlation coefficient on the y-axis. From the outmost left ‘Cell 1’ to ‘Cell 2’, ‘Cell 3’, and outmost right ‘Cell 4’.

Table S1. Key resources

**Reagent type (species) or resource**	**Designation**	**Source or reference**	**Identifiers**	**Additional information**
Genetic reagent (Drosophila melanogaster)	*UAS-jRCaMP1b*	Bloomington Drosophila Stock Center	BDSC: 63793; FLYB: FBti0180189 RRID: BDSC_63793	*PBac{20XUAS-IVS-NES-jRCaMP1b-p10}VK0005;* (Dana et al., 2016)
Genetic reagent (Drosophila melanogaster)	*UAS-GCaMP6s*	Bloomington Drosophila Stock Center	BDSC: 42746 FLYB: FBti0151344 RRID:BDSC_42746	*P{20XUAS-IVS-GCaMP6s}attP40*
Genetic reagent (Drosophila melanogaster)	*UAS-GCaMP6s*			*Sco/CyO; UAS-GCamp6s/TM6b,Tb*; shared by Craig Montell
Genetic reagent (Drosophila melanogaster)	*esgGAL4*	Kyoto DGRC	DGRC: 112304; FLYB: FBti0033872	FlyBase symbol: *w[*]; P{w[+mW.hs]=GawB}NP0726/CyO*
Genetic reagent (Drosophila melanogaster)	*LeAop2-jRCaMP1b*	Bloomington Drosophila Stock Center	BDSC: 64428 FLYB: FBti0181971 RRID: BDSC_64428	*P{13XLexAop2-IVS-NES-jRCaMP1b-p10}su(Hw)attP5*
Genetic reagent (Drosophila melanogaster)	*lexA-esg*	Bloomington Drosophila Stock Center	BDSC: 66632 FLYB: FBti0185046 RRID: BDSC_66632	*P{ST.lexA::HG};* l(3)*/TM6b, Tb (Kockel *et al.*, 2016)
Genetic reagent (Drosophila melanogaster)	*mexGAL4*	Shared by Carl Thummel	FLYB: FBgn0004228	
Genetic reagent (Drosophila melanogaster)	*prosGAL4*	Shared by Sarah Siegrist	FLYB: FBgn0004595	(Matsuzaki *et al.*, 1992)
Genetic reagent (Drosophila melanogaster)	*GBE-Su(H)-GFP::nls*	PMID: 22522699		*w?; mw, GBE-Su(H)-GFP::nls/(CyO); Dr/TM6B* -- from (de Navascués et al., 2012) shared by Joaquin de Navascues
Genetic reagent (Drosophila melanogaster)	*UAS-his2b::CFP*	PMID: 28450412		*w; UAS-his2b::CFP/(CyO); +* -- shared by Yoshihiro Inoue
Genetic reagent (Drosophila melanogaster)	*esg-GFP*	Shared by Norbert Perrimon	FLYB: FBrf0239545	*esg-GFP[KI]/CyO*
Chemical compound	L-glutamic monosodium salt	Alfa Aesar	AAJ6342409	55 mM final concentration
Chemical compound	Trehalose	Sigma Aldrich	T5251-10G	50 mM final concentration
Chemical compound	N-acetyl cysteine	Sigma Aldrich	A9165-5G	2 mM final concentration
Chemical compound	Tri-sodium citrate	Sigma Aldrich	PHR1416-1G	1 mM final concentration
Chemical compound	HEPES	Sigma Aldrich	H0887-20ML	5 mM final concentration

Journal of Cell Science • Supplementary information

Reagent type (species) or resource	Designation	Source or reference	Identifiers	Additional information
Chemical compound, drug	Isradipine	Fisher Scientific, Selleck Chemical LLC	50-153-5018; 50-136-1495	10 µg/m final concentration
Chemical compound, drug	Carbenoxolone	Sigma Aldrich	C4790	100 µM final concentration
Chemical compound	Poly-l-lysine	Sigma Aldrich	P4832-50ML	
Software, algorithm	Fiji		RRID: SCR_002285	Fiji (Schindelin et al., 2012), Bio Formats plugin
Software, algorithm	Icy			Icy (de Chaumont et al., 2012), Active Contours
Software, algorithm	Matlab	Mathworks	RRID: SCR_001622	2019b, Signal Processing Toolbox

Table S2. Average oscillation frequencies per midgut for enterocytes (*mex-GAL4>UAS-GCaMP6s* and *mex-GAL4>UAS-jRCaMP1b*). Each row corresponds to the same recording, i.e., the same movie was used to count oscillations in both interstitial and copper cells. When included in the calculation, non-oscillating cells were given a value of 0.

Sensor	Average oscillation frequency (mHz ± SEM)			
	Including non-oscillating cells		Excluding non-oscillating cells	
	ICs	CCs	ICs	CCs
UAS-GCaMP6s	61 ± 1.3	5.7 ± 2.7	N/A	14 ± 5.0
	15 ± 4.0	6.8 ± 0.7	17 ± 4.0	6.8 ± 0.7
	41 ± 3.1	4.6 ± 1.1	N/A	5.6 ± 1.0
	11 ± 1.4	6.8 ± 1.8	11 ± 1.4	9.4 ± 1.7
	27 ± 0.7	2.2 ± 0.6	N/A	3.8 ± 0.7
UAS-jRCaMP1b	5.3 ± 1.1	3.1 ± 0.8	6.1 ± 1.1	3.7 ± 0.8
	17 ± 2.7	6.8 ± 1.5	N/A	8.1 ± 1.4

Table S3. Average oscillation frequencies per midgut for enteroendocrine cells (*pros-GAL4>UAS-GCaMP6s*) and progenitors (*esgLexA>LexAop-jRCaMP1b*). When included in the calculation, non-oscillating cells were given a value of 0.

Average oscillation frequency (mHz ± SEM)			
Including non-oscillating cells		Excluding non-oscillating cells	
EEs	Progenitors	EEs	Progenitors
9.7 ± 3.0	13 ± 2.3	11.1 ± 3.1	13 ± 2.3
25 ± 5.0	3.8 ± 1.3	25 ± 5.0	6.1 ± 1.6
26 ± 3.7	10 ± 1.5	26 ± 3.7	12 ± 1.0
6 ± 1.9	1.7 ± 0.4	9.1 ± 2.3	2.7 ± 0.4
9.3 ± 2.0	N/A	9.3 ± 2.0	N/A

Journal of Cell Science • Supplementary information

Table S4. Cross correlation coefficient ($Corr_{E,p}$) for all enterocyte and progenitor pairs for time lags up to 12 s. Enterocytes and progenitors are identified by the acquisition channel.

Time lag (s)	Enterocyte 1				Enterocyte 2			
	Progenitor 1	Progenitor 2	Progenitor 3	Progenitor 4	Progenitor 1	Progenitor 2	Progenitor 3	Progenitor 4
**0.000**	0.064	0.128	0.001	-0.045	0.172	0.032	-0.108	0.125
**1.300**	0.010	0.105	0.037	-0.028	0.147	-0.005	-0.071	0.130
**2.600**	-0.009	0.077	0.048	-0.012	0.131	-0.030	-0.047	0.127
**3.890**	-0.029	0.072	0.033	0.006	0.135	-0.049	-0.058	0.115
**5.190**	-0.037	0.042	0.025	0.004	0.141	-0.070	-0.058	0.092
**6.490**	-0.029	0.002	0.012	-0.010	0.143	-0.087	-0.058	0.069
**7.790**	-0.030	-0.025	0.009	-0.013	0.131	-0.083	-0.053	0.051
**9.090**	-0.010	-0.036	0.039	0.011	0.147	-0.089	-0.043	0.033
**10.380**	0.032	0.009	0.070	0.018	0.166	-0.077	-0.026	0.039
**11.680**	0.026	-0.005	0.101	0.027	0.170	-0.088	-0.005	0.039
**Time lag (s)**	**Enterocyte 3**				**Enterocyte 4**			
	**Progenitor 1**	**Progenitor 2**	**Progenitor 3**	**Progenitor 4**	**Progenitor 1**	**Progenitor 2**	**Progenitor 3**	**Progenitor 4**
**0.000**	0.118	0.339	-0.101	0.040	0.050	-0.036	-0.207	0.188
**1.300**	0.141	0.382	-0.088	0.054	0.079	-0.065	-0.216	0.154
**2.600**	0.150	0.419	-0.096	0.093	0.069	-0.112	-0.227	0.153
**3.890**	0.113	0.414	-0.068	0.139	0.053	-0.131	-0.231	0.160
**5.190**	0.092	0.391	-0.047	0.162	0.021	-0.138	-0.203	0.152
**6.490**	0.094	0.333	-0.036	0.182	0.002	-0.163	-0.152	0.139
**7.790**	0.077	0.295	-0.024	0.200	0.003	-0.176	-0.131	0.143
**9.090**	0.062	0.279	-0.022	0.198	0.009	-0.161	-0.130	0.141
**10.380**	0.057	0.215	-0.030	0.196	0.014	-0.173	-0.134	0.144
**11.680**	0.059	0.183	-0.045	0.214	0.002	-0.191	-0.148	0.140

Journal of Cell Science • Supplementary information

Table S5. List of movies analyzed and their related information, including genotype.

Cell type	# z-slices	Spacing (µm)	Condition	Z-plane analyzed	Duration analyzed (s)	# of cells analyzed	Genotype
Copper cell (CCs)	4	4	-	2	539	16	*mexGAL4/esg-LexA>LexAop-jRCaMP1b; UAS-GCaMP6s*
	6	4	-	4	719	11	*mexGAL4/esg-LexA>LexAop-jRCaMP1b; UAS-GCaMP6s*
	6	4	-	6	719	21	*mexGAL4/esg-LexA>LexAop-jRCaMP1b; UAS-GCaMP6s*
	6	4	-	1	293	15	*mexGAL4/esg-LexA>LexAop-jRCaMP1b; UAS-GCaMP6s/Dr*
	9	4	-	2	360	11	*mexGAL4/esg-LexA>LexAop-jRCaMP1b; UAS-GCaMP6s/Dr*
	5	4	CBX	2	719	10	*mexGAL4/CyO; UAS-GCaMP6s*
	6	4	CBX	1	719	12	*mexGAL4/CyO; UAS-GCaMP6s*
	6	4	CBX	4	719	12	*mexGAL4/CyO; UAS-GCaMP6s*
	6	4	CBX	1	1437	10	*mexGAL4/CyO; UAS-GCaMP6s*
Interstitial cell (ICs)	4	4	-	4	539	10	*mexGAL4/esg-LexA>LexAop-jRCaMP1b; UAS-GCaMP6s*
	6	4	-	5	719	12	*mexGAL4/esg-LexA>LexAop-jRCaMP1b; UAS-GCaMP6s*
	6	4	-	4	719	17	*mexGAL4/esg-LexA>LexAop-jRCaMP1b; UAS-GCaMP6s*
	6	4	-	4	293	15	*mexGAL4/esg-LexA>LexAop-jRCaMP1b; UAS-GCaMP6s/Dr*
	9	4	-	7	360	11	*mexGAL4/esg-LexA>LexAop-jRCaMP1b; UAS-GCaMP6s/Dr*
	5	4	CBX	2	719	9	*mexGAL4/CyO; UAS-GCaMP6s*
	6	4	CBX	2	719	12	*mexGAL4/CyO; UAS-GCaMP6s*
	6	4	CBX	2	719	7	*mexGAL4/CyO; UAS-GCaMP6s*
	6	4	CBX	2	1437	10	*mexGAL4/CyO; UAS-GCaMP6s*
Entero-endocrine cell (EEs)	5	2	-	MAX	720	8	*UAS-GCaMP6s/CyO; prosGAL4*
	4	4	-	MAX	495	6	*UAS-GCaMP6s/CyO; prosGAL4*
	6	4	-	MAX	716	7	*UAS-GCaMP6s/esg-LexA>LexAop-jRCaMP1b; prosGAL4/Dr*
	4	4	-	MAX	717	15	*UAS-GCaMP6s/CyO; prosGAL4*
	5	2	-	MAX	720	9	*UAS-GCaMP6s/CyO; prosGAL4*
	5	2	CBX	MAX	720	7	*UAS-GCaMP6s/CyO; prosGAL4*
	6	4	CBX	MAX	719	12	*UAS-GCaMP6s/CyO; prosGAL4*
	4	4	CBX	MAX	360	8	*UAS-GCaMP6s/CyO; prosGAL4*
	3	4	CBX	MAX	720	10	*UAS-GCaMP6s/CyO; prosGAL4*
	4	4	CBX	MAX	357	14	*UAS-GCaMP6s/CyO; prosGAL4*
Progenitors	6	4	-	MAX	430	8	*esg-GAL4>UAS-his2b::CFP, GBE-Su(H)-GFP.nls/CyO; UAS-jRCaMP1b*
	5	2	-	MAX	720	16	*esg-GAL4>UAS-his2b::CFP, GBE-Su(H)-GFP.nls; UAS-jRCaMP1b*
	4	4	-	MAX	717	14	*esg-GAL4>UAS-his2b::CFP, GBE-Su(H)-GFP.nls/CyO; UAS-jRCaMP1b*
	5	2	-	MAX	720	21	*esg-GAL4>UAS-his2b::CFP, GBE-Su(H)-GFP.nls; UAS-jRCaMP1b*
	6	4	CBX	MAX	719	13	*esg-GAL4>UAS-his2b::CFP, GBE-Su(H)-GFP.nls/CyO; UAS-jRCaMP1b*
	6	4	CBX	MAX	719	14	*esg-GAL4>UAS-his2b::CFP, GBE-Su(H)-GFP.nls/CyO; UAS-jRCaMP1b*
	6	4	CBX	MAX	719	12	*esg-GAL4>UAS-his2b::CFP, GBE-Su(H)-GFP.nls; UAS-jRCaMP1b*

Journal of Cell Science • Supplementary information

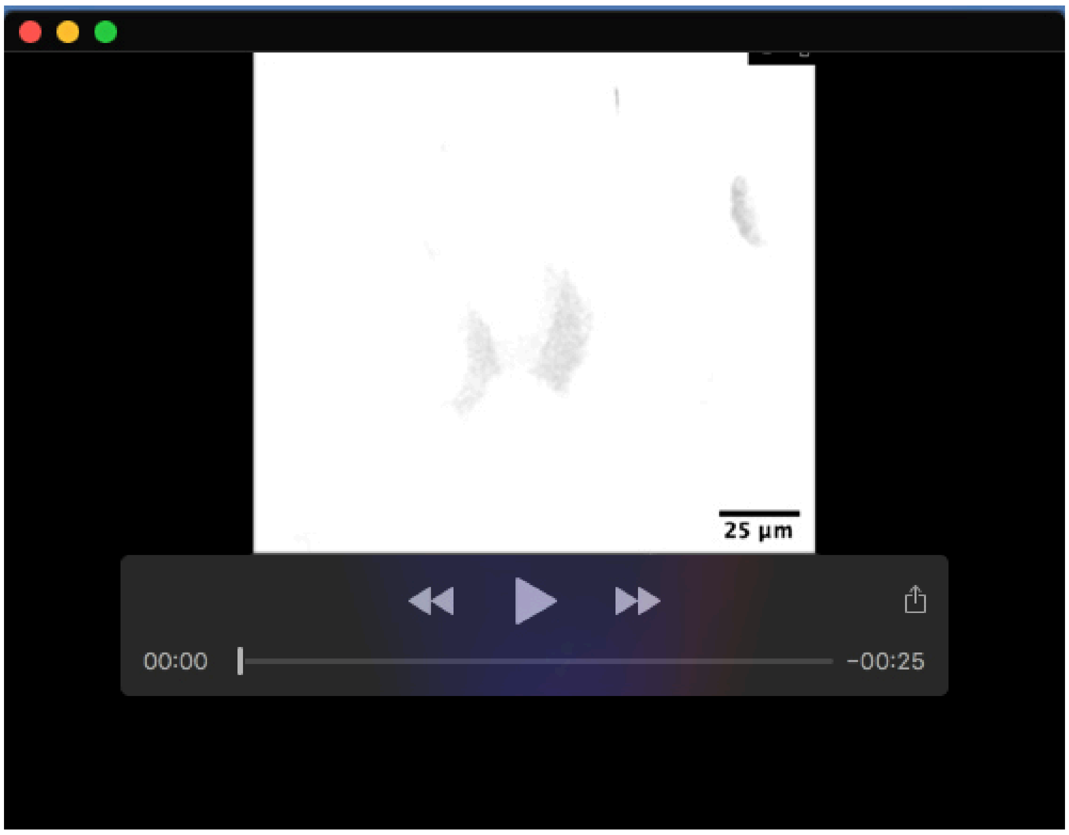

Movie 1. Representative example of long-range calcium waves in enterocytes (interstitial cells, *mex-GAL4>UAS-GCaMP6s*) in a single plane. The movie was used to describe calcium waves in Figures 2 and 3. Scale bar is 25 µm. The movie is sped up 10X.

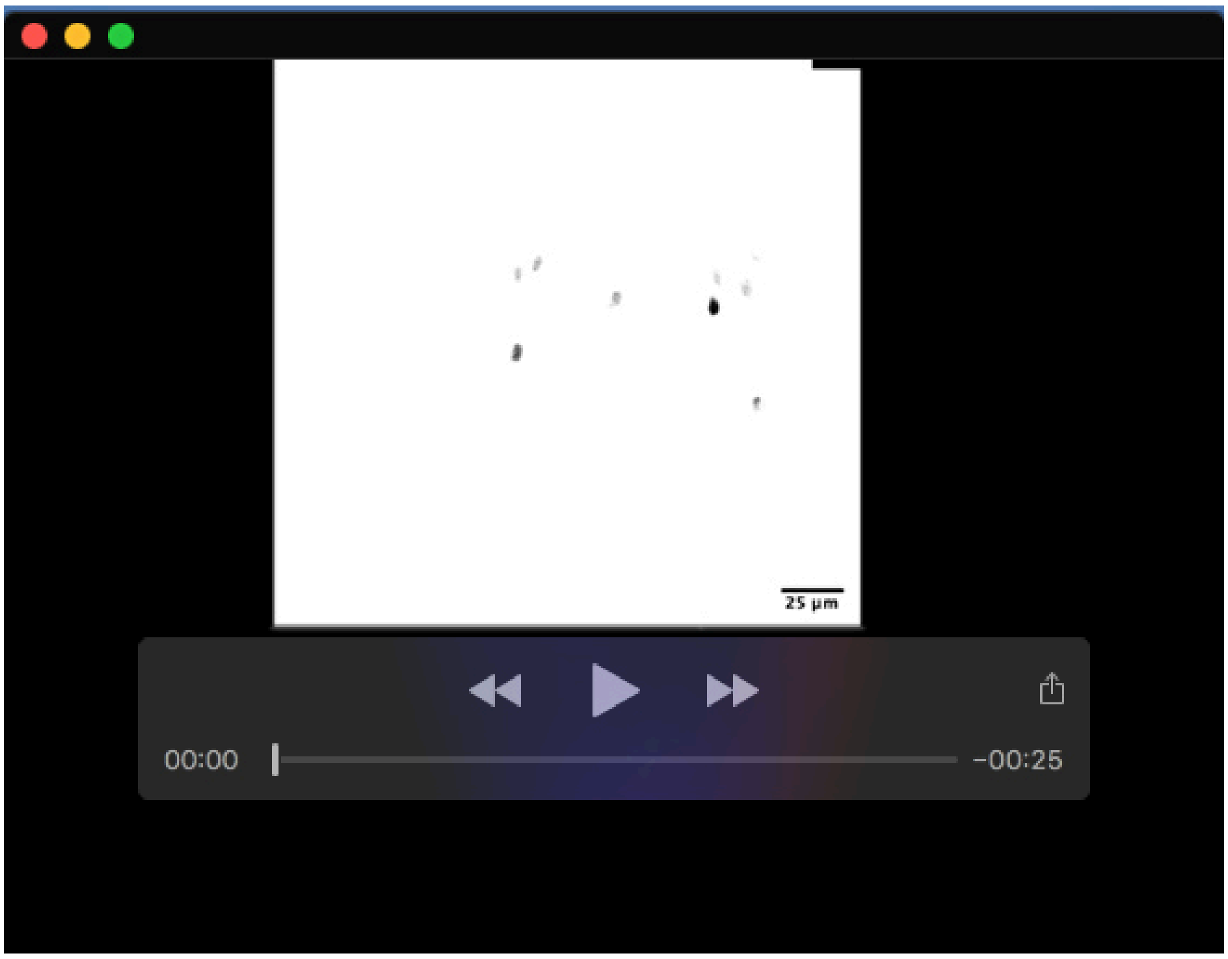

Movie 2. Maximal projection of calcium oscillations in enteroendocrine cells (*pros-GAL4>UAS-GCaMP6s*). Scale bar is 25 µm. Movie is sped up 100X.

Journal of Cell Science • Supplementary information

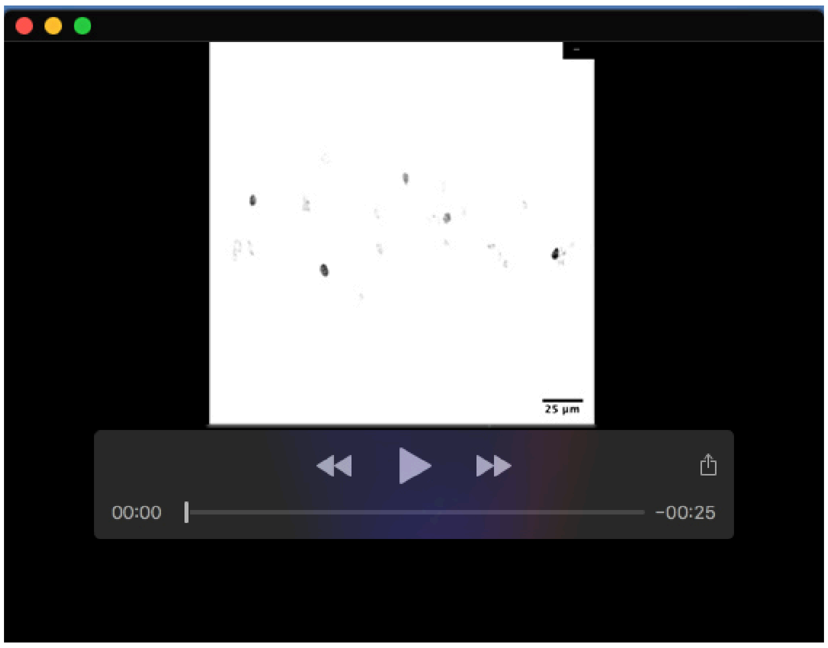

Movie 3. Maximal projection of calcium oscillations in progenitors (*esg-GAL4>UAS-jRCaMP1b*). Scale bar is 25 µm. Movie is sped up 100X.

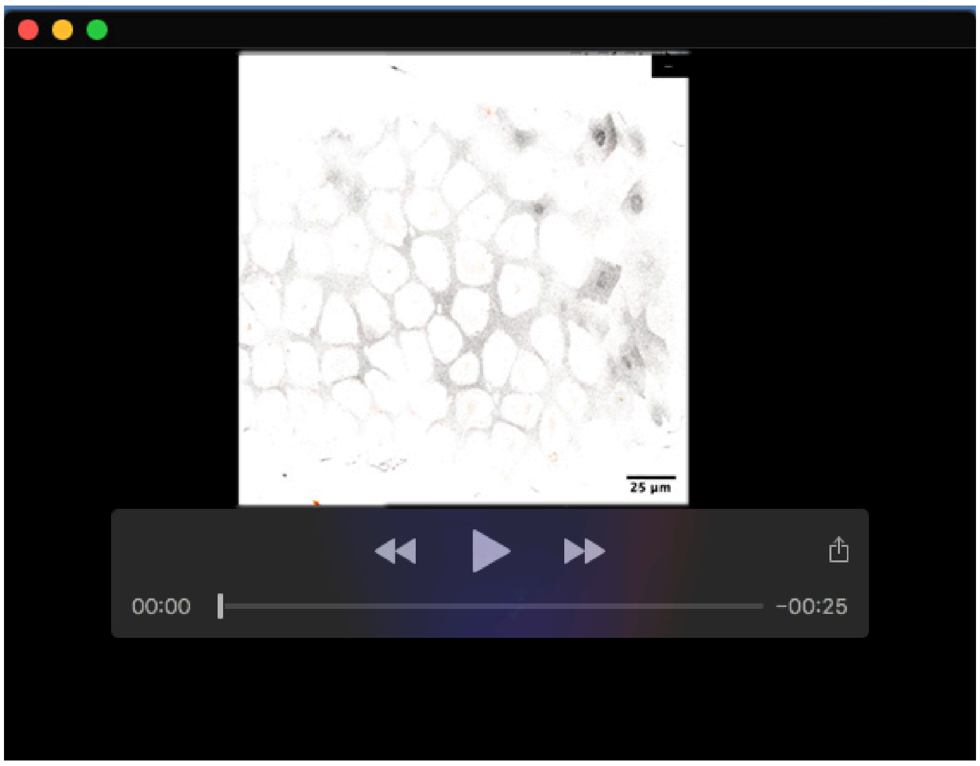

Movie 4. Maximal projection of calcium dynamics in enterocytes (*mex-GAL4>UAS-jRCaMP1b*) and progenitor cells (*esg-LexA>LexAop-jRCaMP1b*), simultaneously. Scale bar is 25 µm. Movie is sped up 10X.

Journal of Cell Science • Supplementary information

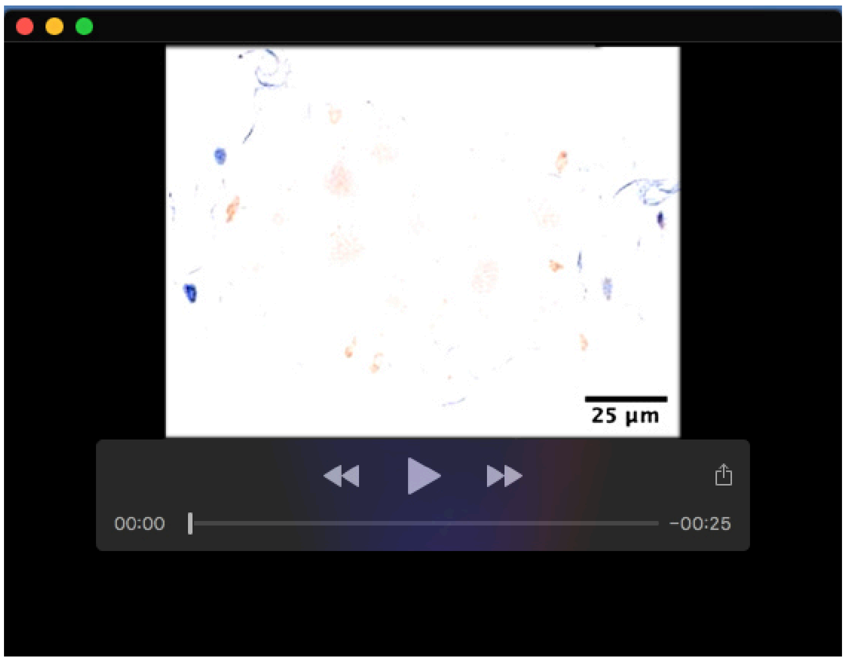

Movie 5. Maximal projection of calcium dynamics in enteroendocrine (*pros-GAL4>UAS-GCaMP6s*) and progenitor cells (*esg-LexA>LexAop-jRCaMP1b*), simultaneously. Scale bar is 25 µm. Movie sped up 10X.

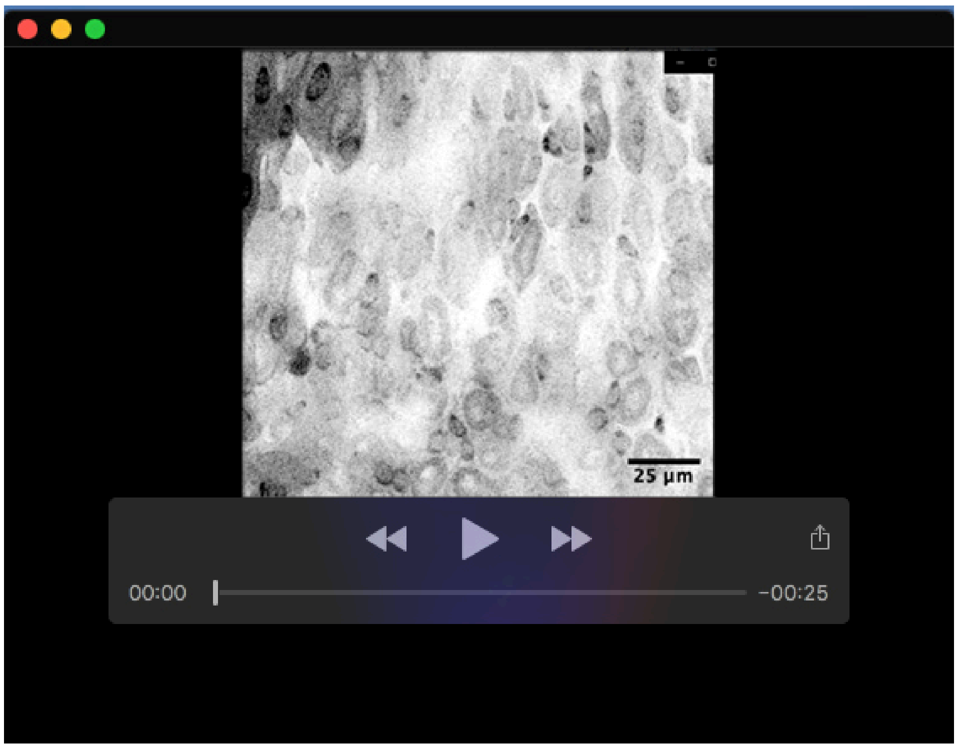

Movie 6. Maximal projection of enterocytes' response (*mex-GAL4>UAS-GCaMP6s*) to the gap junction inhibitor, carbenoxolone (100 µM). Scale bar is 25 µm. Movie is sped up 100X.

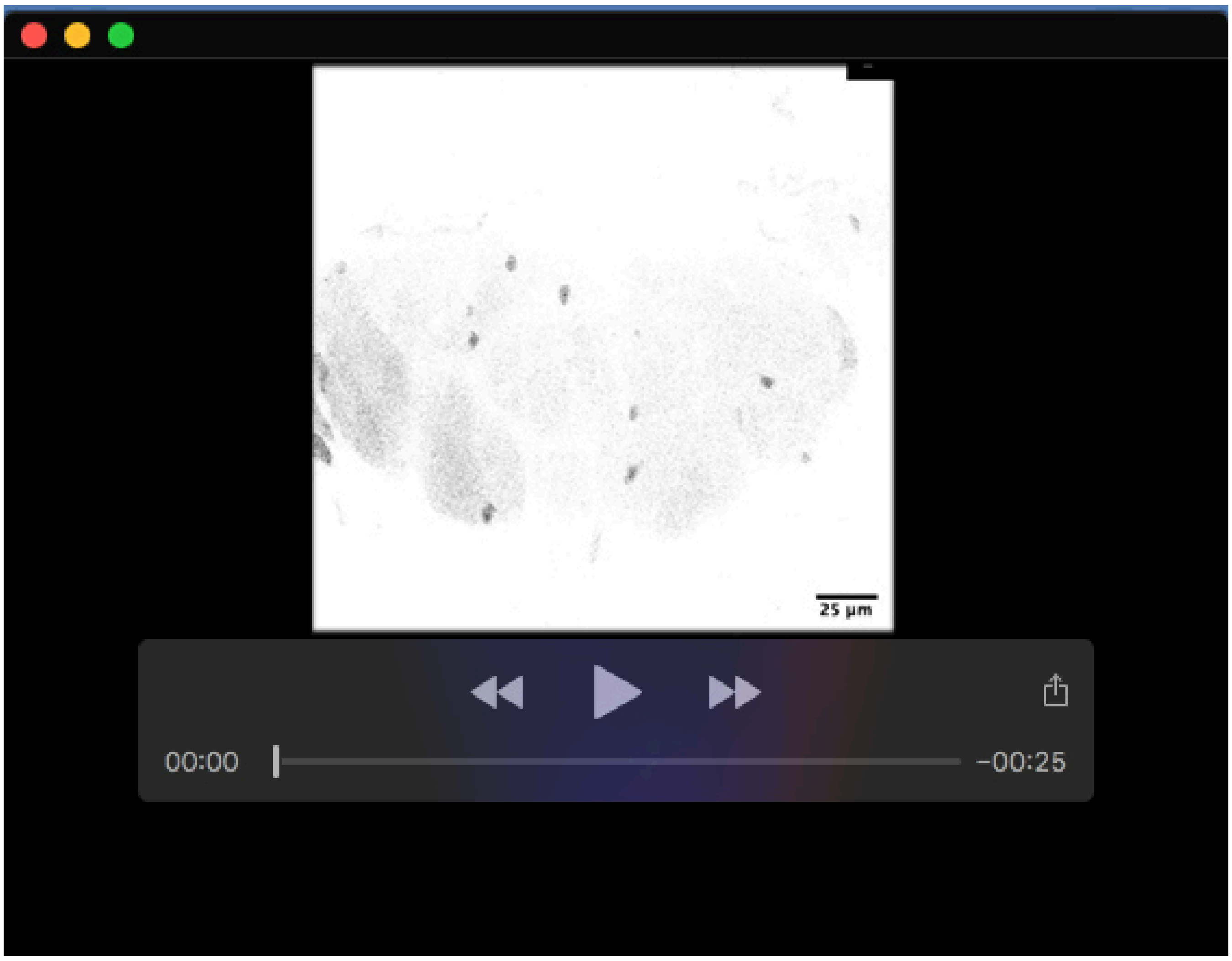

Movie 7. Maximal projection of enteroendocrine cells' (*pros-GAL4>UAS-GCaMP6s*) response to the gap junction inhibitor, carbenoxolone (100 μM). Scale bar is 25 μm. Movie is sped up 100X

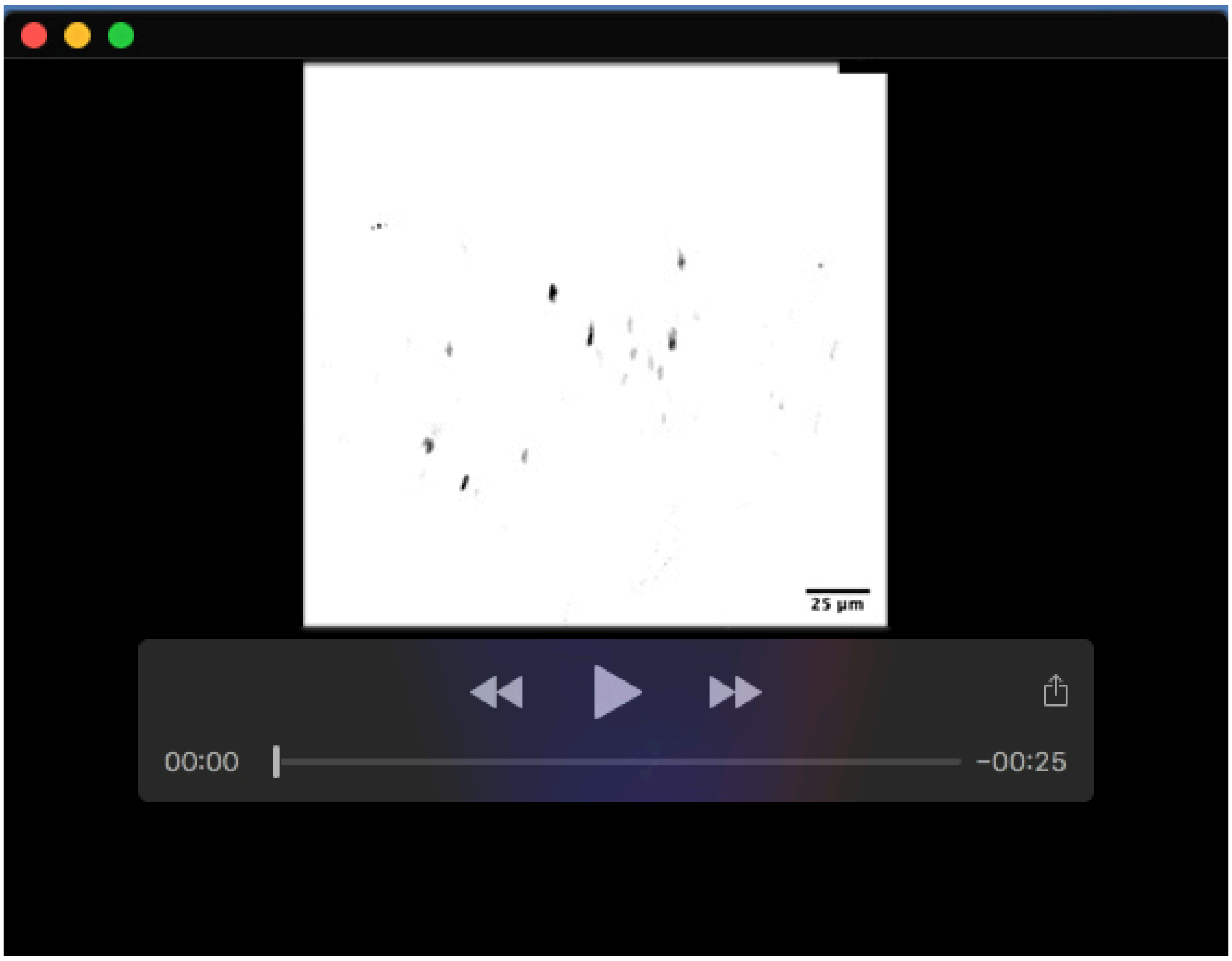

Movie 8. Maximal projection of progenitor cells' (*esg-GAL4>UAS-jRCaMP1b*) response to the gap junction inhibitor, carbenoxolone (100 μM). Scale bar is 25 μm. Movie is sped up 100X.

Journal of Cell Science • Supplementary information
