# Supplementary material for: Independently paced Ca2+ oscillations in progenitor and differentiated cells in an ex vivo epithelial organ
Source: J Cell Sci. 2022 Jul 19;135(14):jcs260249. doi: 10.1242/jcs.260249 (PMC9450890; doi:10.1242/jcs.260249)
Supplement: Reviewer comments [file joces-135-260249_review_history.pdf]

Independently paced Ca^{2+} oscillations in progenitor and differentiated cells in an *ex vivo* epithelial organ

Anna A. Kim, Amanda Nguyen, Marco Marchetti, XinXin Du, Denise J. Montell, Beth L. Pruitt and Lucy Erin O'Brien

DOI: 10.1242/jcs.260249

Editor: John Heath

Review timeline

Submission to Review Commons:	27 October 2021
Original submission:	18 May 2022
Editorial decision:	19 May 2022

Reviewer 1

Evidence, reproducibility and clarity

Review of "independently paced calcium oscillations in progenitor and differentiated cells in an ex vivo epithelial organ"

In this report by Kim et al., the authors describe cell-type specific differences in calcium signaling dynamics in ex vivo cultured adult Drosophila intestines. The interesting findings include: 1. Modular, uncoupled signaling that differs between the multiple cell types in the epithelium; Enteroendocrine cells show single-cell calcium oscillations while enterocytes show long-range calcium waves. Progenitor cells exhibit uncoupled oscillations with half the frequency. The SI video seems to indicate that the waves may be generating some pulses in cell contraction (with a lag) and this could have been discussed. 2. Chemical inhibition of gap junctions with carbenoxolone blocked calcium oscillations for all cell types.

Overall the report was clearly written.

A few points should be addressed to strengthen the results and clarity for the interested reader:
1. There should be a comparison of whether the GCaMP6s and jRCaMP1b report the same frequencies of oscillations to rule out any possible artifacts between sensors. If both sensors are expressed in the same cell is the same frequency result? Is frequency influenced by sensor expression level?
2. It is very surprising that gap junction inhibition blocks even single-cell calcium oscillations. Ref. 18 (published in PLOS CB) reports that gap junction can result in higher levels of single cell oscillations in a different tissue. In that case, it is IP3 dependent. The authors should test if Ca2+ signaling is the gut cell subtypes are IP3 dependent with an PLC inhibitor and IP3R inhibitor. Such experiments should not be more involved than the reported carbenoxolone experiment.
3. P. 7, 8. A statistical test should be performed to quantitatively rule out correlations.
4. P. 9. Typo "prior to to"
5. Are oscillations dependent on Ryr or IP3R?
6. As a calcium channel blocker, is isradipine changing or influencing results? Does carbenoxolone without isradipine still allow for calcium signaling?
7. It is not clear why some of the supplements to the organ culture media are included. No explanation or citation is given (e.g., why is N-Acetyl cysteine added)?
8. Is there any relationship between Ca2+ freq and cell area (between the cell types, which are different sizes)? Or, freq and proliferation rate (i.e., do progenitor cells have a slower cell cycle)
9. Is there a correlation between cell contraction and ca2+ oscillation (perhaps with a lag?)
10. Do progenitors express Inx2 or Inx3 (if this is known)?

© 2022. Published by The Company of Biologists under the terms of the Creative Commons Attribution License (https://creativecommons.org/licenses/by/4.0/).

Minor:

Some formatting issues are present with missing subscripts or superscripts. (e.g., IP3 on p. 2) p. 8 "Figure 4E shows rapid oscillations ~24 mHz in the GCaMP channel and ~20 s wide peaks. (is this a frequency or WHM comparison or a mixed comparison?)

Significance

The report provides a quantitative description of Ca2+ signaling dynamics across multiple subtypes in the Drosophila gut. It reports a surprising finding that gap junction inhibition blocks calcium oscillations, including single-cell oscillations.

Reviewer 2

Evidence, reproducibility and clarity

In this manuscript titled "Independently paced calcium oscillations in progenitor and differentiated cells in an ex vivo epithelial organ", the authors set out to determine calcium dynamics in each cell type of the adult Drosophila intestine by imaging of the whole organ ex vivo. Several interesting observations have been made: each cell type adopts distinct patterns of calcium oscillations; gap junctions mediate calcium oscillations in all three cell types. Interestingly, as the authors indicate, progenitor cells and enteroendocrine cells are only surrounded by enterocytes, but inhibition of gap junctions eliminate calcium oscillations in all three types. This paper describes some interesting observations and provides some mechanistic insights into cell type-specific calcium oscillations. Their data support the conclusions.

Significance

Live calcium imaging of the whole organ represents a significant technical advance. However, it remains unclear what is the functional significance of the fate-specific modes of calcium dynamics. Perhaps, that can be addressed in the further investigation in the future.

Reviewer 3

Evidence, reproducibility and clarity

In this study, Calcium dynamics in ex vivo explant cultures of fly midgut is studied. Some interesting and elegant data are shown: distinct oscillation and spike patterns in different cell types, and waves that appear to propagate in interstitial cells through gap junctions.

These are interesting findings. This manuscript is, however, entirely descriptive. There is not even a hint of the physiological significance of these findings. It is also not clear whether this happens in vivo. The fact that no calcium oscillations are observed in the anterior region may be a physiologically relevant finding, or an artifact of the organ culture preparation. Finally, there may be technical issues. The organ cultures is prepared in the presence of nisradipine, a calcium channel antagonist. This of course raises further doubts regarding physiological significance.

A final minor comment is Fig. 5A, which is purported to show copper cells are calcium sinks. It is not clear how this is demonstrated here.

Significance

The calcium imaging studies are novel, but entirely descriptive. This needs some evidence of functional or physiological relevance, as this may be an in vitro organ culture artifact.

© 2022. Published by The Company of Biologists under the terms of the Creative Commons Attribution License (https://creativecommons.org/licenses/by/4.0/).

Author response to reviewers' comments

1. General Statements [optional]
This section is optional. Insert here any general statements you wish to make about the goal of the study or about the reviews.

Dear Editor:

In this revised manuscript, we demonstrate--for the first time to our knowledge--that calcium waves in a self-renewing, mature epithelial organ (the adult fly gut) undergo dramatic, fate-specific changes as cells differentiate from stem-like into distinct terminal fates. Applying new genetic labeling and granular, 4D quantitative analyses to whole-organ calcium imaging, we discover rapidly swirling calcium waves that propagate rhythmically through one specific terminal cell type--absorptive enterocytes--at the exclusion of other cell types. At the same time, these other cell types (secretory enteroendocrine cells, stem cells) display single-cell calcium oscillations at characteristically distinct frequencies. Multi-cellular enterocyte waves and, to our surprise, single enteroendocrine- and stem-cell oscillations all require gap junctions.

We believe these findings will be of interest to: tissue biologists and physiologists, because of the intriguing implication that particular types of cells in a heterogenous epithelial tissue form electrically coupled networks at the exclusion of other cell types; signal transduction researchers, because of the diverse calcium signaling kinetics occurring simultaneously in the same tissue; and developmental biologists interested in stem cells/fate control, because of the provocative implication that the calcium dynamics of stem cell daughters are continuously set by fate differentiation programs in adult organ renewal.

We must note that due to the departure of key personnel, we are unable to perform additional experiments and thus ask for you to evaluate the manuscript “as is”. Thank you for your consideration; we look forward to your response.

Anna A. Kim and Lucy Erin O’Brien

2. Point-by-point description of the revisions
This section is mandatory. Please insert a point-by-point reply describing the revisions that were already carried out and included in the transferred manuscript.

Summary of major revisions

- New Tables S2 and S3 that report average oscillation frequency per midgut for different cell types and that include average oscillation frequency for enterocytes with jRCaMP1b- sensor.
- New cross correlation analysis between enterocyte and progenitor pairs (Figure S4 and Table S4).
- New analysis of potential correlations between midgut peristaltic contractions and calcium oscillations with a lag (Figures A-C in Point-by-point response below)
- Bioinformatics analysis of midgut cell-type specific expression of innexins (gap junction proteins) in three published transcriptomic datasets.

- New discussion of potential physiological roles for oscillation dynamics in midgut enterocytes and enteroendocrine cells.

Point-by-point response to Reviewer comments

Reviewer #1 (Evidence, reproducibility and clarity (Required)):
Review of "independently paced calcium oscillations in progenitor and differentiated cells in an ex vivo epithelial organ"

In this report by Kim et al., the authors describe cell-type specific differences in calcium signaling dynamics in ex vivo cultured adult Drosophila intestines. The interesting findings include: 1. Modular, uncoupled signaling that differs between the multiple cell types in the epithelium;

© 2022. Published by The Company of Biologists under the terms of the Creative Commons Attribution License (https://creativecommons.org/licenses/by/4.0/).

Enteroendocrine cells show single-cell calcium oscillations while enterocytes show long-range calcium waves. Progenitor cells exhibit uncoupled oscillations with half the frequency. The SI video seems to indicate that the waves may be generating some pulses in cell contraction (with a lag) and this could have been discussed. 2. Chemical inhibition of gap junctions with carbenoxolone blocked calcium oscillations for all cell types.

Overall the report was clearly written.

We thank the reviewer for these comments. We have now performed cross-correlation analysis to examine whether calcium waves and peristaltic contractions are temporally correlated with a lag; please see Point 3 for details. This analysis now discussed in the Results (Lines 181-193).

A few points should be addressed to strengthen the results and clarity for the interested reader:

1a. There should be a comparison of whether the GCaMP6s and jRCaMP1b report the same frequencies of oscillations to rule out any possible artifacts between sensors. If both sensors are expressed in the same cell is the same frequency result?

We have not directly compared the frequencies of the two sensors when co-expressed in the same cell. We have, however, two lines of indirect evidence from us and others indicating that the oscillation frequencies reported by GCaMP6s and jRCaMP1b are comparable:

First, we recorded movies of two *mex>jRCaMP1b* guts and compared them to movies of the five *mex>GCaMP6s* guts that we analyzed in the manuscript. The frequencies of interstitial cell oscillations and copper cell oscillations for each movie are reported in new Table S2 in Supplementary Information (copied below). While oscillation frequencies vary substantially from gut-to-gut for both genotypes, the frequencies exhibited by the *mex>jRCaMP1b* guts generally overlap with those exhibited by the *mex>GCaMP6s* guts.

Second, we note that the frequencies of progenitor cell oscillations that we observe using *jRCaMPb* (ranging from 2.7-13 mHz; please see Table S3 in Supplementary Information) is similar to the frequency of oscillations reported by Deng *Nature* 2015 using *GCaMP3, IVS- GCaMP6s*, and *esg>UAS::tdTomato-2A-GCaMP5* (~5-17 mHz; Deng 2015 Extended Data Figure 5e. Together, these similarities imply that any differences in frequency due to the sensor used are likely insignificant.

We have included an extended version of the data in Supplementary Information (Tables S2 and S3) and added the following sentence to Lines 123-126 of the manuscript:

"The complete dataset of average oscillation frequencies per midgut, including and excluding non-oscillating cells, for all cell types is included in Supplementary Information (Tables S2 and S3)."

Genotype	Mean ICs (mHz ± SEM)	Mean CCs (mHz ± SEM)
mexGal4/esg-LexA>LexAop-jRCaMP1b; UAS-GCaMP6s/Dr	61 ± 1.3	5.7 ± 2.7
mexGal4/esg-LexA>LexAop-jRCaMP1b; UAS-GCaMP6s	15 ± 4.0	6.8 ± 0.7
mexGal4/esg-LexA>LexAop-jRCaMP1b; UAS-GCaMP6s/Dr	41 ± 3.1	4.6 ± 1.1
mexGal4/esg-LexA>LexAop-jRCaMP1b; UAS-GCaMP6s	11 ± 1.4	6.8 ± 1.8

© 2022. Published by The Company of Biologists under the terms of the Creative Commons Attribution License (https://creativecommons.org/licenses/by/4.0/).

mexGal4/esg-LexA>LexAop-jRCaMP1b; UAS-GCaMP6s	27 ± 0.7	2.2 ± 0.6
mexGal4/CyO; UAS- jRCaMP1b	5.2 ± 1.1	3.0 ± 0.7
mexGal4/CyO; UAS- jRCaMP1b	17.0 ± 2.7	6.8 ± 1.5

1b. Is frequency influenced by sensor expression level?

We do not find evidence that oscillation frequency is influenced by level of sensor expression to an extent that is detectable in our system. As one example, we observe that enterocytes in guts containing two copies of *UAS-GCaMP6s* (*mexGAL4*; *UAS-GCaMPs/UAS-GCaMPs*; see Table S5 for complete genotype) express the sensor at visibly higher levels compared to guts that are heterozygous (*mexGAL4*; *UAS-GCaMPs/Dr*; see Table S5 for complete genotype).
Nonetheless, enterocytes with two copies and one copy have comparable oscillation frequencies.

2. It is very surprising that gap junction inhibition blocks even single-cell calcium oscillations. Ref. 18 (published in PLOS CB) reports that gap junction [inhibition??] can result in higher levels of single cell oscillations in a different tissue. In that case, it is IP3 dependent. The authors should test if Ca2+ signaling is the gut cell subtypes are IP3 dependent with an PLC inhibitor and IP3R inhibitor. Such experiments should not be more involved than the reported carbenoxolone experiment.

We were also surprised that single-cell calcium oscillations are blocked by gap junction inhibition. As the Reviewer points out, this outcome contrasts with Soundarrajan *PLOS CB* 2021, which found that inhibiting gap junctions in wing imaginal discs (also using carbenoxolone) increased the frequency of single-cell calcium oscillations. We agree that one would not necessarily expect that single-cell calcium oscillations, which can be regarded as cell-autonomous, would be blocked by inhibiting gap junctions, which are intercellular conduits.

We have not explicitly investigated the function of IP3 in midgut calcium oscillations. However, Deng *Nature* 2015 found that oscillations of single progenitor cells are dependent on progenitor cell expression of IP3R. Since, as we report here, these oscillations are blocked by carbenoxolone, the fact that they are also IP3-dependent suggests that regulation of oscillations in the midgut is different from the wing disc.

One potential explanation for this difference may be distinct involvement of plasma membrane calcium channels. Soundarrajan 2021 and prior studies from the Zartman group (Narciso 2017, Brodskiy 2019) found that in the wing disc, plasma membrane channels are dispensable for intracellular calcium dynamics. By contrast in the midgut, Deng and co-workers (*Nature* 2015) found that the plasma membrane channel PMCA is essential for progenitor cell oscillations. Thus, extracellular calcium is an important contributor to calcium flux in the midgut but not in wing discs.
This difference may underlie why gap junction inhibition increases single-cell oscillations in one tissue and blocks them in another.

3. P. 7, 8. A statistical test should be performed to quantitatively rule out correlations.

We thank the reviewer for this suggestion. We have now quantitatively examined cross correlations between the time series data for enterocytes and progenitors in Figure 4E. In brief, our cross correlation analysis does not support that the oscillations of enterocytes and progenitors are correlated.

We describe the analysis in the Results section (Lines 181-193) as follows:

"To quantitatively assess whether calcium oscillations of progenitors and enterocytes were correlated, we analyzed the four ROIs (Figure 4E) that cover progenitor cells 1-4 and their juxtaposed enterocytes (direct enterocyte-progenitor pairs) by applying a formula for cross

© 2022. Published by The Company of Biologists under the terms of the Creative Commons Attribution License (https://creativecommons.org/licenses/by/4.0/).

correlations between two time series with time lag h (see Supplementary Information, Cross correlation with time lag for finite time series, p. 5). With time lag 0, which corresponds to the Pearson correlation, the enterocyte-to-progenitor cross correlations for the four direct enterocyte-progenitor pairs in the ROIs (enterocyte 1 to progenitor 1, enterocyte 2 to progenitor 2, etc.) have absolute values less than 0.2 for all four direct pairs (these four correlation values are approximately 0.064, 0.032, -0.10, and 0.19), indicating weak correlation. When all non-zero time lags are considered, the cross correlations for direct enterocyte-progenitor pairs never exceed an absolute value of 0.32 (Figure S1). These values do not support that progenitor and enterocyte oscillations are correlated.

We also considered cross correlations between enterocyte-progenitor pairs in which the enterocyte and the progenitor are in different ROIs. Table S4 reports all the correlation values for all direct and non-direct pairs for time lags up to 12 seconds (**Error! Reference source not found.**).”

4. P. 9. Typo "prior to to"

Corrected, thank you.

5. Are oscillations dependent on Ryr or IP3R?

Oscillations of progenitor cells previously were found to depend on IP3R but not RyR (Deng *Nature* 2015). We have not examined whether oscillations of enterocyte or enteroendocrine cell oscillations depend on IP3R or RyR.

6. As a calcium channel blocker, is isradipine changing or influencing results? Does carbenoxolone without isradipine still allow for calcium signaling?

This is a salient question. Isradipine is frequently included in midgut live imaging protocols to minimize peristaltic contractions of the gut’s visceral muscle layer (e.g. Deng *Nature* 2015, Zhang *Dev Cell* 2019, Marchetti *bioRxiv* 2021). The drug is an antagonist of L-type calcium channels, which are expressed in muscle fibers and neurons.

Although we cannot exclude that isradipine-sensitive, L-type channels are expressed in the gut epithelium, we believe that if such channels were primarily responsible for midgut calcium dynamics, then isradipine would result in wholesale blocking of calcium oscillations, akin to the effect of the gap junction inhibitor carbenoxolone. Consistent with this notion, Deng 2015 (Extended Data Fig 5f) found that isradipine (10 µg/ml, which is the same concentration as in our studies) did not alter the frequency of progenitor cell oscillations. In addition, Marchetti 2021 reports that kinetics of progenitor cell differentiation (enteroblasts to enterocytes) in isradipine-containing cultures resemble those inferred from *in vivo* lineage tracing.

In our hands, guts undergo rapid peristalsis when isradipine is omitted. The vigorous movements prevent us from tracking single cells, which unfortunately precludes a direct comparison of calcium oscillations with and without the drug. We did not attempt to perform imaging with carbenoxolone and without isradipine because we would not be able to compare such movies with appropriate control movies lacking both carbenoxolone and isradipine.

7. It is not clear why some of the supplements to the organ culture media are included. No explanation or citation is given (e.g., why is N-Acetyl cysteine added)?

We apologize for the confusion. An optimized media recipe for *ex vivo* midgut culture was developed by one of the co-authors (Marchetti). After our manuscript was submitted to Review Commons, a separate preprint describing this recipe was posted to the *bioRxiv* (Marchetti 2021 https://doi.org/10.1101/2021.12.17.473114). This preprint is now cited in the Results and Methods sections (Lines 98 and 328, respectively).

In brief, N-acetyl cysteine and sodium citrate are antioxidants; they were added to the culture media to reduce phototoxicity during imaging. These roles are now briefly described in the Methods (lines 335 and 337).

© 2022. Published by The Company of Biologists under the terms of the Creative Commons Attribution License (https://creativecommons.org/licenses/by/4.0/).

8. Is there any relationship between Ca2+ freq and cell area (between the cell types, which are different sizes)? Or, freq and proliferation rate (i.e., do progenitor cells have a slower cell cycle)

At a gross level, the frequency of calcium oscillations does not correlate with cell size. Enteroendocrine cells are smaller than copper cells and larger than most diploid progenitors, yet enteroendocrine cells oscillate more frequently than both copper cells and progenitor cells.

The frequency of calcium oscillations also does not correlate with proliferation rate. In this organ, stem cells account for nearly all cell proliferation, whereas copper cells and enteroendocrine cells are post-mitotic. However, progenitor cells (including stem cells (*c.f.* Deng 2015)) oscillate faster than copper cells and slower than enteroendocrine cells.

9. Is there a correlation between cell contraction and ca2+ oscillation (perhaps with a lag?)

We are inferring that the reviewer is asking about peristaltic contractions—i.e. spatiotemorally coordinated contractions of adjacent cells in the gut tube. To examine whether peristaltic contractions correlated with calcium oscillations, we first identified the timing of peristaltic contractions. Contraction events in a selected ROI were visible as a change in brightness in the transmitted-light channel (Figure A). Over the course of the 12-minute movie, contractions became more frequent; this trend is likely due to reduced effectiveness of the muscle paralytic isradipine, which was included in the imaging media (10 µg/ml).

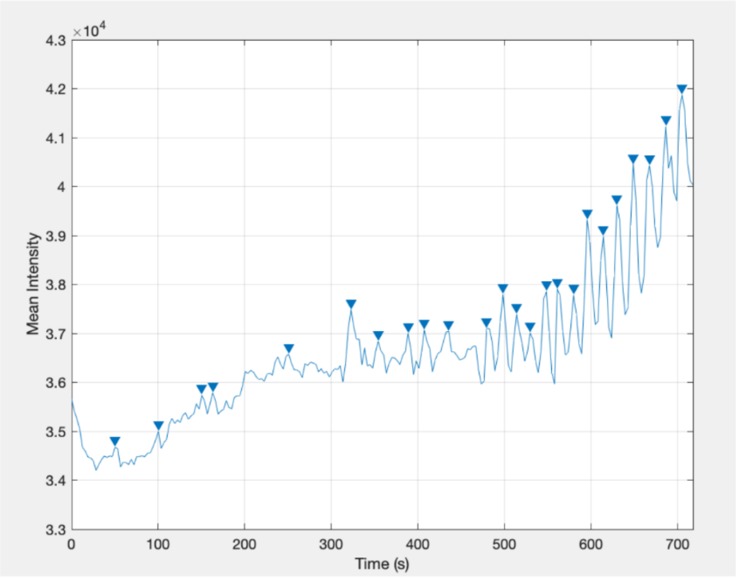

Figure A. Midgut peristaltic contractions over time in a representative movie ROI. Peristaltic contractions (blue arrowheads) are visible in the transmitted light channel as a local peak in intensity. Contraction frequency and baseline intensity increase over time, likely due toreduced effectiveness of the muscle paralytic isradipine, which was included in the imaging media (10 µg/ml).

We next compared the timing of contraction events with timing of calcium oscillations in the same movie. To do this, we measured GCaMP intensity for three individual interstitial cells (Figure B) and four individual copper cells (Figure C) that participated in the peristaltic contractions. Oscillations were measured from t=0-540 seconds in the movie; after t=540 seconds, contractions were too frequent for single cells to be recognized.

Comparing single-cell calcium oscillations to whole-gut peristaltic contractions (blue arrowheads), we observed neither a synchronous nor a lagging temporal correlation. Single calcium oscillations occurred before, at the same time as, and after individual contraction events. In addition, while the frequency of peristaltic contractions increased over time, the frequencies of single-cell calcium oscillations did not. These findings imply no direct relationship between the relative timing of peristaltic contractions and of calcium oscillations in our movies.

© 2022. Published by The Company of Biologists under the terms of the Creative Commons Attribution License (https://creativecommons.org/licenses/by/4.0/).

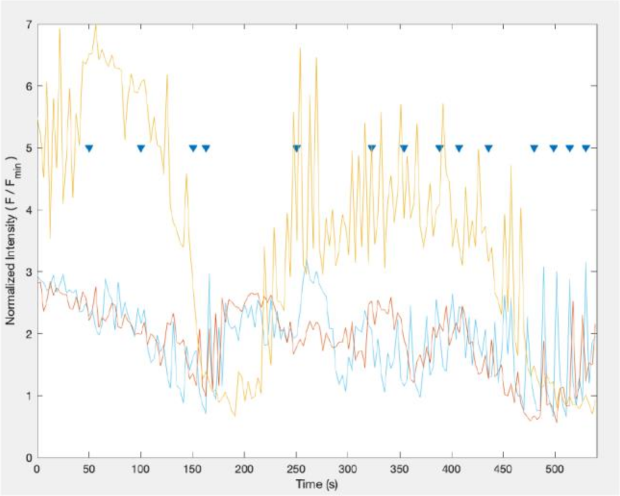

Figure B. Calcium oscillations of interstitial cells do not correlate with peristaltic contractions (blue arrowheads). GCaMP intensities (colored lines) are plotted for three interstitial cells from t=0-540 seconds of the same movie ROI as Figure A. Peristaltic contraction events (*c.f.* Figure A) are shown as blue arrowheads.

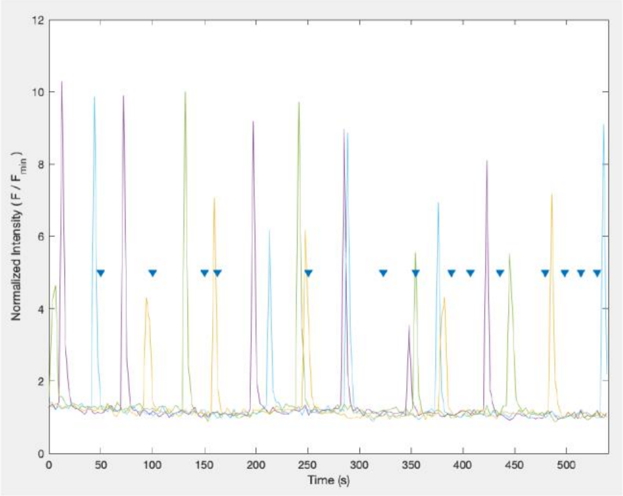

Figure C. Calcium oscillations of copper cells do not correlate with peristaltic contractions (blue arrowheads). GCaMP intensities (colored lines) are plotted for four copper cells from t=0- 540 seconds of the same movie ROI as Figure A. Peristaltic contraction events (*c.f.* Figure A) are shown as blue arrowheads.

10. Do progenitors express Inx2 or Inx3 (if this is known)?

To our knowledge, no studies examining Innexins in the adult *Drosophila* midgut have yet been published. However, three transcriptomic datasets are available (bulk RNAseq: Dutta *Cell Reports* 2015 http://flygutseq.buchonlab.com; single-cell RNAseq: Hung *PNAS* 2020 https://www.flyrnai.org/scRNA/gut/; and single-cell RNA nucseq: Li *Science* 2022 https://flycellatlas.org/). We searched expression of the eight Drosophila *inx* genes in all three datasets. This identified midgut expression of *inx2*, and *inx3* in all three datasets and of *inx7* in the Hung and Li datasets. Cell type-specific expression of these three innexins is as follows:

For enterocytes, the three datasets are generally consistent. Both *inx2* and *inx7* are expressed in enterocytes in the midgut overall. In addition, the Dutta dataset, which contains region- specific analyses, shows *inx2* and *inx7* specifically in R3 enterocytes. Consistent with the R3- specific data from Dutta 2015, the Hung and Li whole-gut datasets (which do not separate out the R3 region)

© 2022. Published by The Company of Biologists under the terms of the Creative Commons Attribution License (https://creativecommons.org/licenses/by/4.0/).

show expression of *inx7* and *inx2* in copper cells, which are exclusively in R3, and broadly in other enterocytes. (R3 Interstitial cells were not explicitly identified in the sc-seq datasets).

For enteroendocrine cells and progenitor cells, the three datasets were less consistent. For enteroendocrine cells, the Dutta R3-specific dataset detects *inx7* and *inx2*, the Li dataset detects *inx2* and *inx3*, whereas the Hung dataset detects *inx3*.

For progenitor cells, the Dutta R3-specific dataset shows expression of *inx2* in stem cells and *inx2* and *inx7* in stem cells and enteroblasts. The Hung and Li datasets show only sporadic expression of *inx2* or *inx7* in progenitors and show widespread expression of *inx3* in progenitors.

One possible explanation for the differences between the Dutta R3-specific dataset and the Hung and Li whole-gut datasets is that innexin expression in R3 may be different compared to the midgut overall. Technical differences in the three studies (for instance, use of bulk RNAseq vs single-cell RNAseq vs single-cell RNA nucseq) may also underlie these differences. An interesting objective for the future will be to assess cell type-specific protein expression and localization of the three midgut innexins and to perform functional studies to assess their cell- specific contributions to calcium dynamics.

Minor:

Some formatting issues are present with missing subscripts or superscripts. (e.g., IP3 on p. 2)

We thank the reviewer and have corrected the formatting issues.

p. 8 "Figure 4E shows rapid oscillations ~24 mHz in the GCaMP channel and ~20 s wide peaks. (is this a frequency or WHM comparison or a mixed comparison?)

We apologize for the confusion. Our intention was to highlight the characteristic calcium dynamics for the different cell types within the same recording using the dual gene expression system. To clarify, we have revised the sentence as follows (Lines 176-180):

“We illustrate the feasibility of using the orthogonal driver systems to simultaneously image calcium dynamics in interstitial cells and progenitors. Characteristic, ~24 mHz oscillations are observed for interstitial cells in the GCaMP channel and wide signal elevations (20 s) in the RCaMP channel; these are comparable to observations when using a single driver system (**Error! Reference source not found.**D).”

Reviewer #2 (Evidence, reproducibility and clarity (Required)):

In this manuscript titled "Independently paced calcium oscillations in progenitor and differentiated cells in an ex vivo epithelial organ", the authors set out to determine calcium dynamics in each cell type of the adult Drosophila intestine by imaging of the whole organ ex vivo. Several interesting observations have been made: each cell type adopts distinct patterns of calcium oscillations; gap junctions mediate calcium oscillations in all three cell types. Interestingly, as the authors indicate, progenitor cells and enteroendocrine cells are only surrounded by enterocytes, but inhibition of gap junctions eliminate calcium oscillations in all three types. This paper describes some interesting observations and provides some mechanistic insights into cell type-specific calcium oscillations.
Their data support the conclusions.

Reviewer #2 (Significance (Required)):

Live calcium imaging of the whole organ represents a significant technical advance. However, it remains unclear what is the functional significance of the fate-specific modes of calcium dynamics. Perhaps, that can be addressed in the further investigation in the future.

We thank the Reviewer for their comments.

© 2022. Published by The Company of Biologists under the terms of the Creative Commons Attribution License (https://creativecommons.org/licenses/by/4.0/).

Reviewer #3 (Evidence, reproducibility and clarity (Required)):

In this study, Calcium dynamics in ex vivo explant cultures of fly midgut is studied. Some interesting and elegant data are shown: distinct oscillation and spike patterns in different cell types, and waves that appear to propagate in interstitial cells through gap junctions.

These are interesting findings. This manuscript is, however, entirely descriptive. There is not even a hint of the physiological significance of these findings.

We agree that identifying the functions of enterocyte and enteroendocrine oscillations is an important goal. Although we do not identify these functions here, our work opens an avenue for such studies in the future. We have now added discussion of what these roles may be. Based on studies in tissues such as *Drosophila* imaginal discs, the possibilities include actomyosin organization, occluding junction remodeling, mechanosensation, and control of organ-scale processes such as growth and defecation. This new paragraph encompasses Lines 256-276.

It is also not clear whether this happens in vivo. The fact that no calcium oscillations are observed in the anterior region may be a physiologically relevant finding, or an artifact of the organ culture preparation.

The focus of this manuscript is on calcium dynamics in the R3 region; we do not draw conclusions about other midgut regions. We have added a sentence to the text that states whether the anterior R2 region exhibits calcium oscillations in vivo is unknown (Lines 106-108): "We did not observe any calcium dynamics in the anterior (R2) region, which may reflect a physiological difference in this region of the gut, or which may be a consequence of dissection or *ex vivo* culture.".

Finally, there may be technical issues. The organ cultures is prepared in the presence of isradipine, a calcium channel antagonist. This of course raises further doubts regarding physiological significance.

We thank the Reviewer for raising this point. Isradipine is often included in midgut live imaging protocols to minimize peristaltic contractions of the gut's visceral muscle layer (e.g. Deng *Nature* 2015, Zhang *Dev Cell* 2019, Marchetti *bioRxiv* 2021). In our hands, guts undergo rapid peristalsis when isradipine is omitted. The vigorous movements prevent us from tracking single cells, which unfortunately precludes us from assessing the potential effects of isradipine on calcium oscillations.

Isradipine is an antagonist of L-type calcium channels, which are expressed in muscle fibers and neurons. While we cannot exclude that isradipine-sensitive, L-type channels are expressed in the gut epithelium, we believe that if such channels were primarily responsible for midgut calcium dynamics, then isradipine would result in wholesale blocking of these dynamics, akin to the effect of the gap junction inhibitor carbenoxolone. Consistent with this notion, Deng 2015 (Extended Data Fig 5f) found that isradipine did not alter the frequency of progenitor cell oscillations. In addition, Marchetti 2021 reports that kinetics of progenitor cell differentiation (enteroblasts to enterocytes) in isradipine-containing cultures resemble those inferred from *in vivo* lineage tracing.

A final minor comment is Fig. 5A, which is purported to show copper cells are calcium sinks. It is not clear how this is demonstrated here.

We apologize for the confusion about Figure 5A. We speculate that carboenoxolone treatment of the gut causes copper cells to accumulate cytosolic calcium (e.g. behave as calcium sinks). The reasoning is as follows:

Copper cells switch from having extremely low baseline GCaMP signal in untreated guts (e.g., Fig. 1B and D, Fig. 2A-E, Fig. 3C and 3D, Fig. 4C and 4D) to having high baseline GCaMP signal in carbenoxolone-treated guts. Conversely, interstitial cells switch from having high, dynamic GCaMP signal in homeostatic guts to having low, mostly static GCaMP signal in carbenoxolone-treated guts.

These two complementary effects--loss of calcium oscillations in interstitial cells and concomitant acquisition of high, static calcium in copper cells--are consistent with a scenario in which gap junction inhibition causes copper cells to accumulate cytosolic calcium at the expense of interstitial

© 2022. Published by The Company of Biologists under the terms of the Creative Commons Attribution License (https://creativecommons.org/licenses/by/4.0/).

cells—i.e. that copper cells are behaving as a "calcium sink".

We hope that our revised text explains this point more clearly (Lines 219-222):

"One possible explanation for this unexpected result is that copper cells may accumulate cytosolic calcium, perhaps from extracellular sources, when calcium flux through interstitial cells is blocked."

Reviewer #3 (Significance (Required)):

The calcium imaging studies are novel, but entirely descriptive. This needs some evidence of functional or physiological relevance, as this may be an in vitro organ culture artifact.

Prior studies (Deng *Nature* 2015, He *Nature* 2018) have reported that Ca^{2+} oscillations in midgut progenitors regulate two physiologically relevant cell behaviors: stem cell divisions and enteroendocrine differentiation. The progenitor Ca^{2+} oscillations that we observe *ex vivo* have frequencies similar to those reported in the Deng and He studies and thus are likely to have the same functional roles.

We believe that the Ca^{2+} oscillations we observe in enterocytes and enteroendocrine cells *ex vivo* likely reflect similar dynamics *in vivo*. Identifying the functions of enterocyte and enteroendocrine oscillations remains an important goal for future work. In the current manuscript, we have added new discussion on what these functions may be, extrapolating from known functions of calcium oscillations in other epithelial systems and physiological roles of midgut enterocytes and enteroendocrine cells. (Lines 256-276).

Original submission

First decision letter

MS ID#: JOCES/2022/260249

MS TITLE: Independently paced calcium oscillations in progenitor and differentiated cells in an ex vivo epithelial organ

AUTHORS: Anna A Kim, Amanda Nguyen, Marco Marchetti, Denise J Montell, Beth L Pruitt, and Lucy E O'Brien
ARTICLE TYPE: Research Article

Thank you for sending your manuscript to Journal of Cell Science through Review Commons.

I am happy to tell you that your manuscript has been accepted for publication in Journal of Cell Science, pending standard ethics checks.

© 2022. Published by The Company of Biologists under the terms of the Creative Commons Attribution License (https://creativecommons.org/licenses/by/4.0/).
